# Impact of Module-X2 and Carbohydrate Binding Module-3 on the catalytic activity of associated glycoside hydrolases towards plant biomass

**DOI:** 10.1038/s41598-017-03927-y

**Published:** 2017-06-16

**Authors:** Nandita Pasari, Nidhi Adlakha, Mayank Gupta, Zeenat Bashir, Girish H. Rajacharya, Garima Verma, Manoj Munde, Rakesh Bhatnagar, Syed Shams Yazdani

**Affiliations:** 1 Microbial Engineering Group, International Centre for Genetic Engineering and Biotechnology, Aruna Asaf Ali Marg, New Delhi, India; 2 DBT-ICGEB Centre for Advanced Bioenergy Research, International Centre for Genetic Engineering and Biotechnology, Aruna Asaf Ali Marg, New Delhi, India; 3 Transcription Regulation group, International Centre for Genetic Engineering and Biotechnology, Aruna Asaf Ali Marg, New Delhi, India; 4 School of Biotechnology, Jawaharlal Nehru University, New Delhi, India; 5 School of Physical Sciences, Jawaharlal Nehru University, New Delhi, India

## Abstract

Cellulolytic enzymes capable of hydrolyzing plant biomass are secreted by microbial cells specifically in response to the carbon substrate present in the environment. These enzymes consist of a catalytic domain, generally appended to one or more non-catalytic Carbohydrate Binding Module (CBM), which enhances their activity towards recalcitrant biomass. In the present study, the genome of a cellulolytic microbe *Paenibacillus polymyxa* A18 was annotated for the presence of CBMs and analyzed their expression in response to the plant biomass and model polysaccharides Avicel, CMC and xylan using quantitative PCR. A gene that encodes X2-CBM3 was found to be maximally induced in response to the biomass and crystalline substrate Avicel. Association of X2-CBM3 with xyloglucanase and endoglucanase led to up to 4.6-fold increase in activity towards insoluble substrates. In the substrate binding study, module X2 showed a higher affinity towards biomass and phosphoric acid swollen cellulose, whereas CBM3 showed a higher affinity towards Avicel. Further structural modeling of X2 also indicated its potential role in substrate binding. Our findings highlighted the role of module X2 along with CBM3 in assisting the enzyme catalysis of agricultural residue and paved the way to engineer glycoside hydrolases for superior activity.

## Introduction

Cellulose, hemicellulose, and lignin are interlinked in different proportions to constitute lignocellulosic biomass, the most abundant storehouse of naturally fixed carbon^1^. Efficient breakdown of this recalcitrant biomass into monomeric sugar is a major limiting step in the economical production of second generation biofuel^2^. The quasi-crystalline structure of cellulose is known to limit the action of cellulolytic enzymes^3^. To overcome this limitation, certain cellulolytic enzymes possess a non-catalytic Carbohydrate Binding Module (CBM) capable of binding to the insoluble polysaccharide substrate and bringing it in a close proximity to the Catalytic Domain (CD)^4–6^. CBMs are a contiguous stretch of amino acids present within the Carbohydrate Active Enzyme (CAZyme) and are grouped into 80 families on the basis of sequence similarity in the CAZy database^7^ till date. Though members of each family show structural similarity, notable variation is observed in substrate specificity. CBMs can be present in single, tandem or multiple copies within the modular structure of CAZymes^7^. Same CBM present in tandem leads to increased avidity whereas different CBMs when together allow binding to different substrates or substructures^8^.

A considerable reduction in hydrolytic potential was noted upon truncation of the CBM from the corresponding enzyme, suggesting a critical role of CBMs in substrate recognition and hydrolysis^9–12^. They not only enhance proximity of CD to the substrate^13^ but also increase the penetration abilities of the enzyme into the substrate^14^. Furthermore, Thongekkaew *et al*.^15^ and Telke *et al*.^16^ reported enhancement of binding affinity and hydrolytic activity of a given cellulolytic enzyme upon fusion with an extra CBM. Structure-function based rational approaches have also been employed to improve the action of the cellulolytic enzymes on crystalline substrates^17^. However, biomass hydrolysis is still considered to be the most expensive step in the bioethanol production process and therefore there is a need to strategize systematic approaches to improvise enzymatic action on the recalcitrant components of the lignocellulosic biomass. The identification of appropriate CBM(s) that enhances the ability of different glycoside hydrolases to act on their crystalline substrates by such systematic approach can lead to a consequent reduction in the cost of biomass degradation^2, 3^. In the current study, we have devised a strategy to identify CBM(s) capable of binding to lignocellulosic biomass, based on the well-studied phenomenon of increased expression of hydrolytic enzymes in presence of a cellulosic substrate^18–21^.

In the earlier study, we identified *P*. *polymyxa* A18 as a promising cellulose degrading bacterium amongst the ones screened from the guts of termite, pill-bug, and rice stem borer^22^. It not only yielded the maximum amount of cellulases but its overall ability to degrade biomass was also found to be higher. Driven by these factors, it served as a potential host platform in the current study for the identification of CBMs that could enhance biomass hydrolysis process. We thus analyzed the genome of *P*. *polymyxa* A18 for the presence of genes encoding potential CBMs. The expression of the polypeptides containing these CBMs was monitored through quantitative PCR in response to pretreated biomass and crystalline & amorphous carbon substrates. A polypeptide containing X2-CBM3 consisting of module X2 and CBM3 in tandem showed maximum induction in response to pretreated biomass and crystalline carbon substrate. To further investigate the role of this protein stretch, the catalytic module was expressed separately and in association with individual modules X2 and CBM3 and tested for biomass hydrolysis. Since biomass hydrolyzing potential of the native enzyme was greater than its truncated counterparts, the role of these modules was further investigated by detecting the affinity of each of them including tandem X2-CBM3 towards pretreated biomass. Phylogenetic analysis and structural modeling of module X2 were also performed to understand its diversity and role in substrate recognition. Moreover, the polypeptide containing X2-CBM3 was expressed in association with hydrolytic enzymes, endoglucanase and xylanase, which inherently possessed minimal hydrolytic activity towards the biomass. This study not only indicated the importance of CBMs in biomass hydrolysis but also provided insights into how CBM engineering could be deployed to improve the binding affinity of cellulolytic enzymes and hence their hydrolytic potential.

## Results

### Identification of Carbohydrate Binding Modules (CBMs) in *P*. *polymyxa* A18 genome

**Figure 1:**
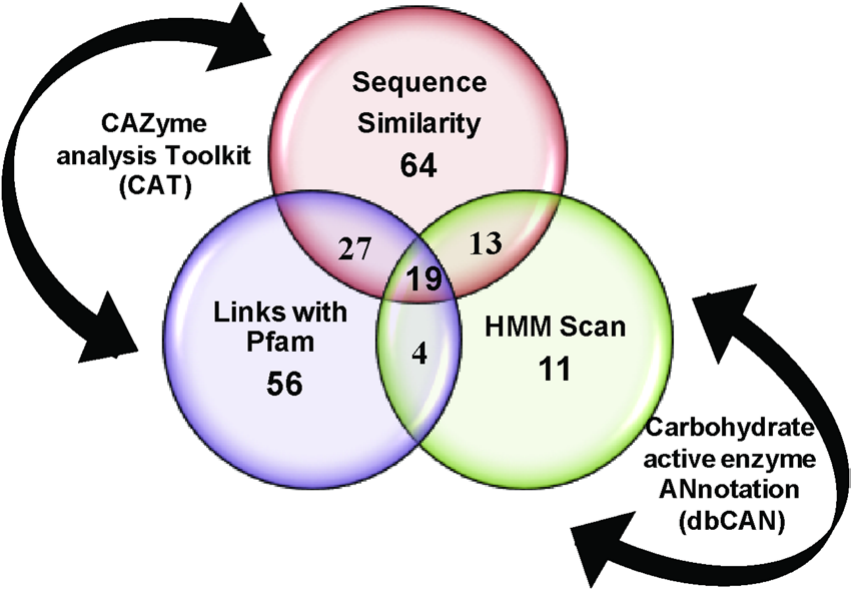
Venn diagram representing the number of CBMs predicted by different approaches in the genome of *P*. *polymyxa* A18. Detailed analysis is provided in Supplementary Table S1.

**Figure 2:**
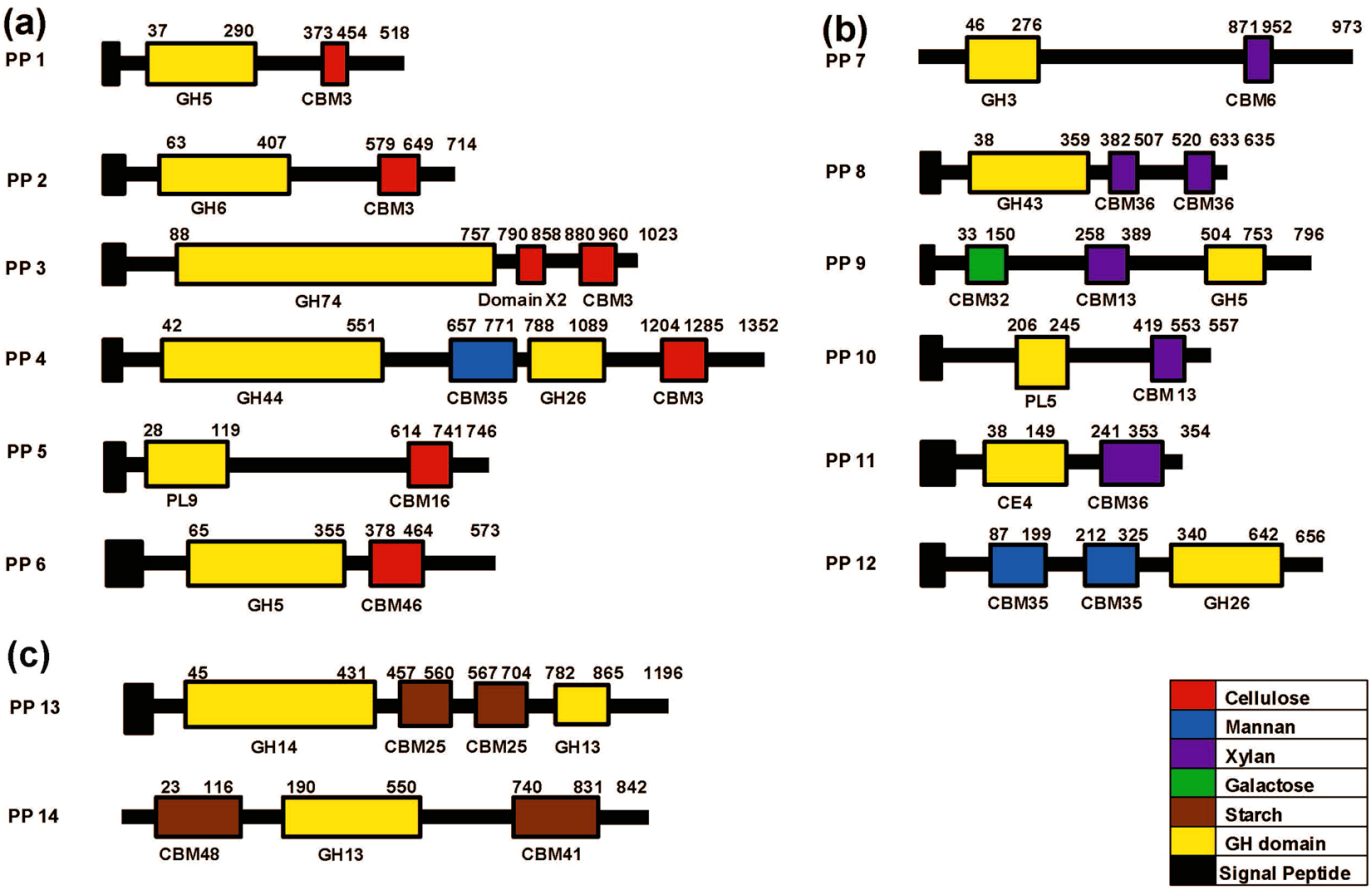
Domain architecture of CBM containing polypeptides identified in *P*. *Polymyxa* A18 genome. The numbering above the polypeptides shows the amino acid positions of the module boundaries as determined by HMM scan search. CBMs are color coded according to the previous knowledge on their binding affinity and categorized on the basis of (**a**) cellulose binding, (**b**) hemicellulose binding and (**c**) starch binding. Black and yellow boxes represent a potential signal sequence and the catalytic domain, respectively.

In order to identify CBMs present in *P*. *polymyxa* A18 genome, its genome sequence was annotated using RAST server. Predicted proteins were screened for CBMs using Carbohydrate active enzyme ANnotation (dbCAN)^23^ and CAZymes Analysis Toolkit (CAT)^24^. 46 CBMs were predicted using dbCAN whereas 121 and 103 CBMs were predicted using CAT based on sequence similarity and linkage of CAZy families with protein family domains (Pfam), respectively. Since there were variations in the number of CBMs predicted, for greater accuracy, CBMs predicted were compared and only the ones identified throughout were used for further experimental analysis (Fig. 1 and Supplementary Table S1). A consensual data set comprising of 19 CBMs localized in 14 polypeptides was identified through the analysis. In order to understand the association of catalytic modules with each of the CBM, dbCAN results were further used to visualize the architecture of these polypeptides (Fig. 2 and Table 1). The CBM containing polypeptides were categorized into three types based on the existing knowledge of binding affinity of CBM towards cellulose, hemicellulose, and starch, respectively (Fig. 2a–c). It should be noted that these annotations included two amylase encoding polypeptides which were not predicted to have a signal sequence (PP7 and PP14). They were still included in the study because these were mere bioinformatic based predictions. CBM3, which is capable of binding to cellulose^25^, was found in abundance and associated with a variety of glycoside hydrolase (GH) domains; such as GH5 and GH44 with endoglucanase activity, GH6 with cellobiohydrolase activity, GH26 with mannanase activity, and GH74 with xyloglucanase activity (Table 1). Some other interesting CBM arrangements were also observed. Seven polypeptides contained more than one CBM in them; amongst which PP3 contained module X2 along with CBM3 (Fig. 2a). PP4 contained two CBMs each, following their respective GHs, and thus appeared to be a fusion of two genes as was predicted earlier for similar polypeptide^26^. PP9 contained two CBMs, CBM32 and CBM13, known to bind diverse substrates, i.e., polygalactouronic acid^27^ and xylan^28^, respectively, which were associated with GH5. PP13 contained two catalytic domains belonging to families GH13 and GH14, both predicted for amylase activity. dbCAN results showed the presence of Polysaccharide Lyase module (PL) in PP5 and PP10, but they were not detected by CAT analysis. CBM25, CBM35 and CBM36 were present as tandem repeats within their modular polypeptides. Despite extensive functional and structural studies of CBMs being performed, the consequences of the fusion of a given CBM to a specific GH are yet unpredictable^17, 29^. We performed further studies to better understand the association of CBMs with a given CD. This would eventually lead to the identification of potential CBM(s) that would assist CDs in enhancing biomass hydrolysis.

**Table 1:** CBM-containing polypeptides of *P*. *Polymyxa* A18 used for expression analysis.

PP	CBM	GH	NCBI ID	Function of the closest characterized homolog	Ref.
1.	3	GH5	WP_026065408.1	Endoglucanase	17
2.	3	GH6	WP_016821398.1	Cellobiohydrolase	71
3.	3, X2	GH74	WP_017427741.1	Xyloglucanase	35
4.	3, 35	GH26, GH44	WP_017427072.1	Endoglucanase, xylanase, lichenase, Mannanase	26
5.	16	PL9	WP_017426818.1	Uncharacterized	
6.	46	GH5	WP_038978163.1	Endoglucanase	72
7.	6	GH3	WP_017428582.1	β-Glucan glucohydrolase	73
8.	36, 36	GH43	WP_017426918.1	Arabinoxylan arabinofuranohydrolase	74
9.	13, 32	GH5	WP_017426982.1	Uncharacterized	
10.	13	PL5	WP_017426909.1	Uncharacterized	
11.	36	CE4	WP_017426884.1	Xylanase deacetylase	75
12.	35, 35	GH26	WP_017428011.1	β–mannanase	76
13.	25, 25	GH14, GH13	WP_017426122.1	α-amylase	77
14.	48, 41	GH13	WP_017426749.1	Type I pullulanase	78

### Identification of CBMs expressed in response to biomass

All the CBMs considered in our study were found to be associated with one or more carbohydrate active catalytic domain as predicted by dbCAN results. Production and secretion of carbohydrate active enzymes by a cellulolytic microbe depend solely on the type of carbon substrate present in the surrounding medium^30–33^. We have shown in our earlier report that amongst all the gut microbes grown on alkali-treated biomass, *P*. *polymyxa* A18 produced most efficient enzymes for biomass hydrolysis^22^. In order to identify the CBMs, which play a role in biomass hydrolysis, we studied the expression level of 14 genes that encode CBM containing polypeptides using quantitative real-time PCR (qRT-PCR). Ammonium hydroxide-treated wheat straw was used as biomass (will be referred to as biomass) to grow *P*. *polymyxa* A18; compositional analysis of this insoluble biomass suggests that it is primarily composed of polysaccharides made of glucose and xylose (see Methods section). The pre-treated biomass is mostly heterogeneous in nature containing both crystalline and amorphous polysaccharides^34^. In order to detect the impact of these components of biomass in the expression of genes for CBM containing polypeptides, we included Avicel PH101 (a commonly used polymer of glucose with crystallinity index ranging from 74–84% and degree of polymerization as 480)^35^, CMC low viscosity (a chemically derived amorphous polymer of glucose with degree of polymerization as 400) and beechwood xylan (a polymer of xylose with degree of polymerization as 30)^36^ as carbohydrate substrates in the study.

**Figure 3:**
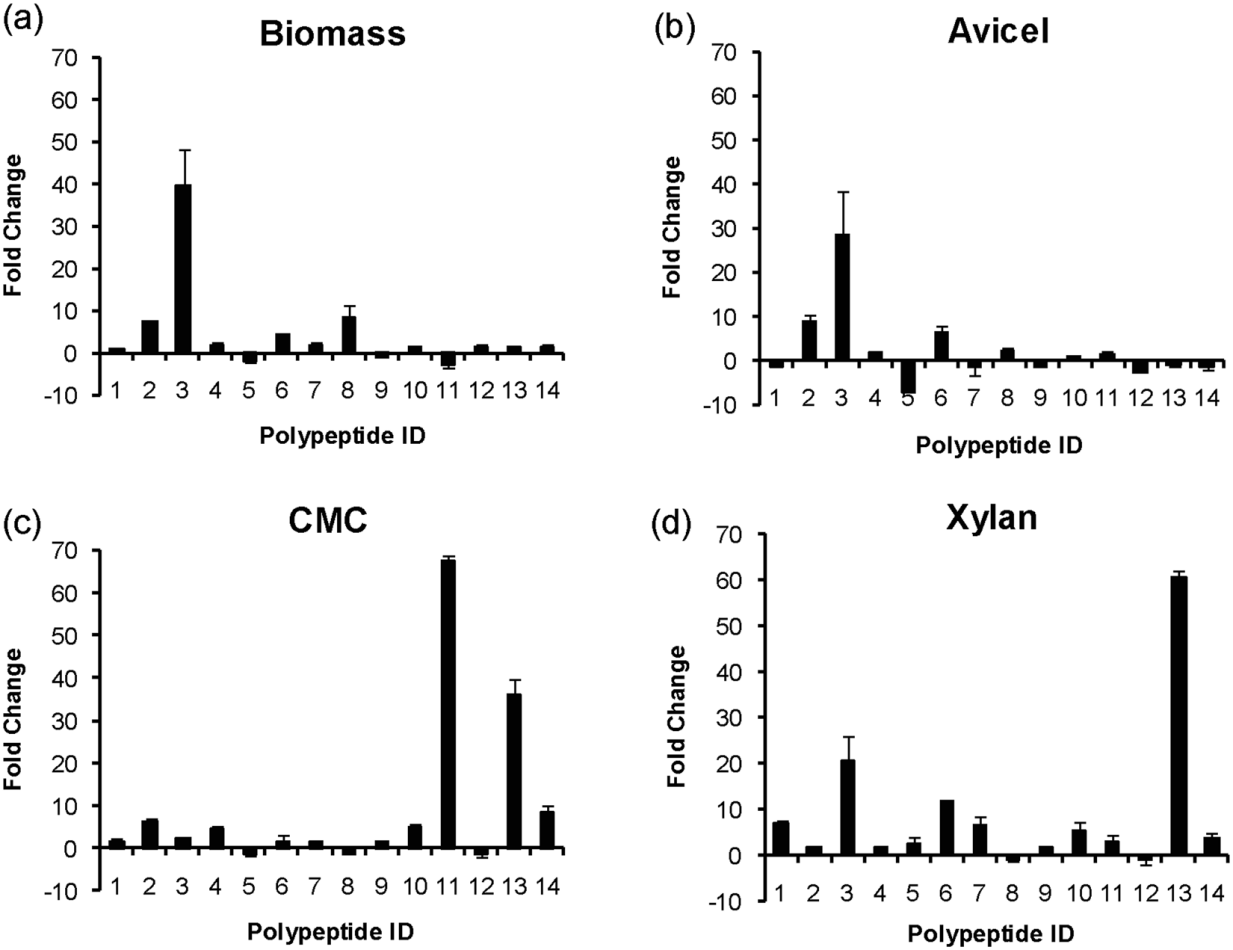
Relative transcript level of CBM containing polypeptides during growth on different carbon substrates. *P*. *Polymyxa* A18 was grown in presence of (**a**) ammonium hydroxide-treated biomass, (**b**) Avicel, (**c**) CMC and (**d**) xylan, and transcript levels of 14 CBM-containing polypeptides were estimated using qRT-PCR. Fold change was calculated with respect to *P*. *Polymyxa* A18 grown without the carbon substrate and normalized on 16S rRNA. Error bars represent standard error of mean calculated over four data obtained from two technical replicates of each of two biological replicates.

As a prerequisite to this study, we examined the growth and cellulolytic activity of *P*. *polymyxa* A18 on all the above-stated carbon substrates (Supplementary Fig. S1). The growth was found to be optimal at 8 hr for all the substrates used (Supplementary Fig. S1a), and hence cells were harvested at this time point for RNA isolation. qRT-PCR was performed to determine the expression levels of the genes encoding these polypeptides in response to different carbon substrates. Relative expression values were calculated using Livak method^37^ wherein 16S rRNA was used as an internal control gene. Fold change was calculated with respect to relative expression values of the gene obtained from cells grown without any carbon substrate (Fig. 3). The fold change values with p-value < 0.05 were considered significant during further analysis (Supplementary Table S2).

Transcript for PP3, which exhibits xyloglucanase activity got maximally induced (~40-fold) in the presence of biomass (Fig. 3a and Supplementary Table S2). Other polypeptides whose transcripts were significantly induced by the biomass were for PP8 possessing arabinoxylan arabinofuranohydrolase activity (8.2 fold), PP2 possessing cellobiohydrolase activity (7.7 fold), and PP6 containing endocellulase activity (4.6 fold) (Fig. 3a). The polypeptides induced in response to biomass reflected the importance of the carbohydrate active catalytic domains associated with these polypeptides in the hydrolysis of various components of biomass. Interestingly, PP3 was also highly transcribed (~29-fold) in the case of Avicel, followed by PP2 (9 fold) and PP6 (6.4 fold) (Fig. 3b). CMC majorly induced PP11, PP13 and PP14 having hemicellulase and amylase activity in addition to PP2, PP4 and PP10 having cellulase and lyase activity (Fig. 3c). Xylan also induced polypeptides containing cellulase (PP1 and PP6), hemicellulase (PP3), amylase (PP13) and lyase (PP10) activity (Fig. 3d), indicating that both xylan and CMC act as an inducer for a number of diverse enzymes.

Amongst all the polypeptides, PP3 showed remarkably high expression in the presence of biomass and Avicel. The domain structure of PP3 suggested that it has tandem modules of X2 and CBM3 downstream of the GH74 family catalytic domain, which possesses xyloglucanase activity (Fig. 2a). Considering that this polypeptide gets induced mainly by the crystalline substrate, i.e., Avicel, and the insoluble substrate, i.e., biomass, it was imperative to further explore the role of X2-CBM3 in assisting the associated catalytic module in hydrolysing its substrate.

### Impact of X2-CBM3 on the activity of associated xyloglucanase

**Figure 4:**
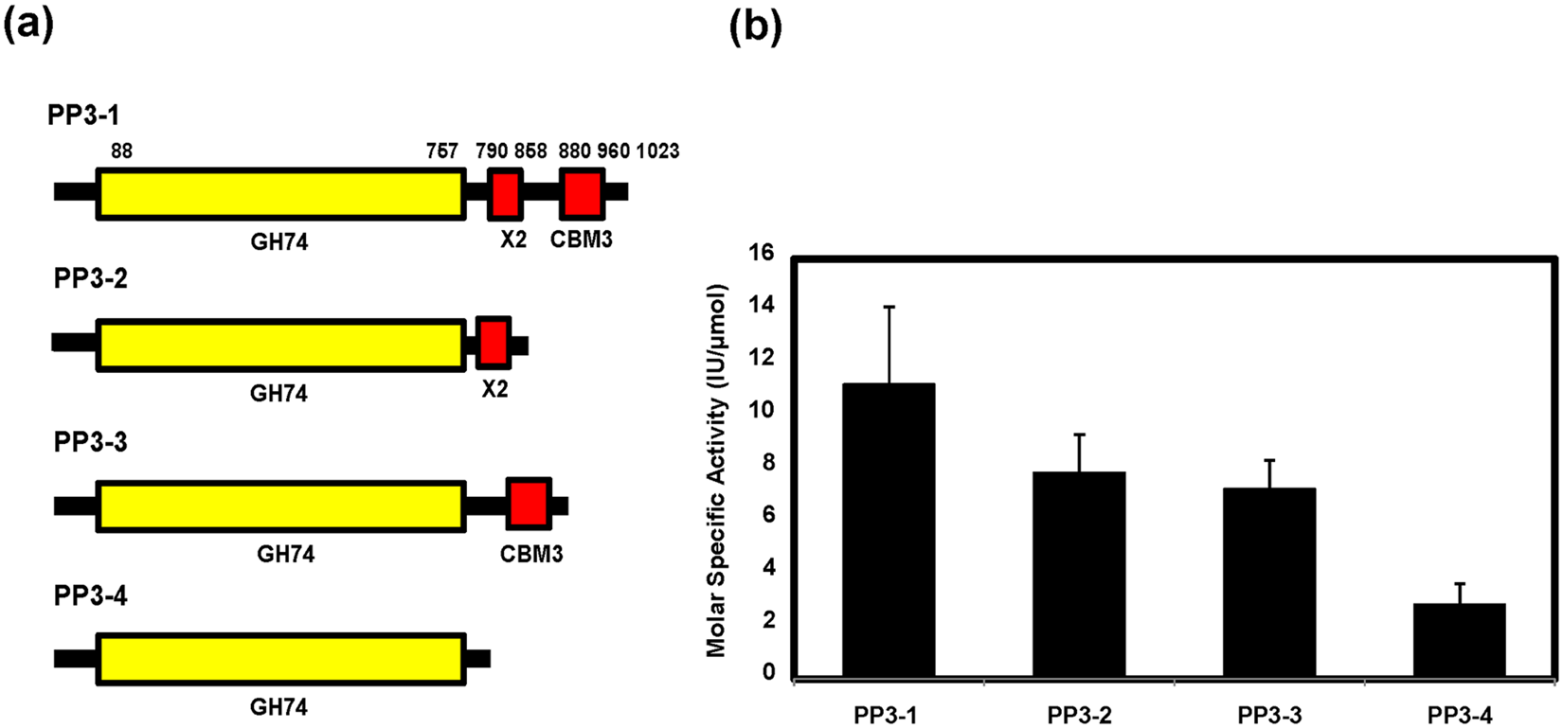
(**a**) Molecular organization of X2-CBM3 containing xyloglucanase and its truncated derivatives used in the study and (**b**) Specific activity of the derivatives against ammonia-treated biomass. Error bars represent the standard deviations of experiments performed in duplicate.

To explore the role of module X2 and CBM3 in the catalytic function of xyloglucanase present in PP3 polypeptide, we made following constructs (Fig. 4a) – (i) construct PP3-1 containing GH74 along with module X2 and CBM3, (ii) construct PP3-2 containing GH74 domain along with only module X2, (iii) construct PP3-3 containing GH74 domain along with only CBM3, and (iv) construct PP3-4 containing only GH74 catalytic domain. All the constructs were expressed without their native signal sequence and along with a 6-his tag at the N-terminus to facilitate affinity purification. Heterologous expression was carried out in *E*. *coli*, proteins were purified by immobilized metal affinity chromatography, and their catalytic activities were determined using its native substrate xyloglucan^35^. Since PP3 was highly induced by the biomass that is known to contain xyloglucan tightly bound with cellulose microfibril through hydrogen bonding^38, 39^, we wanted to analyze the role of CBMs in assisting catalytic domain (CD) to hydrolyze this biomass as well. The X2-CBM3 seemed to improve the catalytic activity of xyloglucanase towards the soluble substrate xyloglucan by 2.5-fold (Supplementary Table S3). The individual modules had a minor impact on the activity. In order to compare the cleavage pattern of all the four variants of PP3, the digestion products of xyloglucan were analyzed using HPLC and mass spectrometry. Three major peaks belonging to XLLG, XLXG/XXLG and XXXG oligomers (Supplementary Fig. S2) were identified, which resembled the product profiles of xyloglucanase of the GH74 family reported before^35^. The results also indicated that truncation had no impact on the enzyme specificity (Supplementary Fig. S2). When the enzyme activity was tested on the insoluble biomass, the full-length enzyme (PP3-1) displayed 4.6-fold higher activity on biomass as compared to truncated polypeptide containing only GH74 (PP3-4) (Fig. 4b and Supplementary Table S3), which indicated the importance of X2-CBM3 in crystalline substrate recognition. Association of GH74 CD with either module X2 or CBM3 improved the hydrolysis as compared to GH74 alone; however, it was lower than the full-length xyloglucanase that contained the X2-CBM3 (Fig. 4b). This observation indicated that the association of both module X2 and CBM3 were responsible for achieving optimal hydrolysis of insoluble substrate.

### Binding of X2-CBM3 with soluble and insoluble substrates

After affirming the positive role of X2-CBM3 in assisting the associated xyloglucanase catalytic domain in hydrolysing its substrate, we further examined the binding affinity of this polypeptide and its constituting modules for soluble and insoluble substrates. To perform this experiment, module X2, CBM3 and X2-CBM3 were recombinantly expressed in *E*. *coli* and purified. The binding affinity for the soluble substrate of GH74 xyloglucanase, i.e., tamarind xyloglucan was determined by isothermal calorimetry (ITC) with the help of thermodynamic parameters (ΔH, ΔS, and ΔG) and affinity constant (K_A_) using the single site binding model^40, 41^ (Supplementary Fig. S3 and Table 2). CBM3 displayed significant binding affinity (K_A_ = 11.0 × 10^4^ Mol^−1^) with favorable enthalpy (ΔH =−17.5 kcal/mol) and unfavorable entropy contribution (TΔS = −9.9 kcal/mol) (Table 2). However, binding of X2-CBM3 with xyloglucan showed lower affinity (K_A_ = 4.0 × 10^4^ M^−1^) with the interactions driven mainly by entropy (TΔS = +20.9 kcal/mol). The binding isotherm for module X2 (Supplementary Fig. S3c) seemed to display complex binding and the data could not be fitted using the single site model.

**Table 2:** Binding affinity constants (K_A_) and thermodynamic properties of independent modules and xyloglucan interactions^*^.

Protein	K_A_ × 10^4^ (M^−1^)	ΔH (kcal/mol)	TΔS (kcal/mol)	ΔG (kcal/mol)
X2	nd^ǂ^	nd^ǂ^	nd^ǂ^	nd^ǂ^
CBM3	11.0 ± 0.9	−7.0 ± 0.6	−0.7 + 0.04	−6.3 ± 0.7
X2-CBM3	4.3 ± 0.4	14.6 ± 2.4	20.9 ± 1.1	−6.3 ± 0.4

^*^ITC thermograms are available as supplementary information [see Supplementary Fig. S3].

^ǂ^Could not be determined (explained in the text).

**Figure 5:**
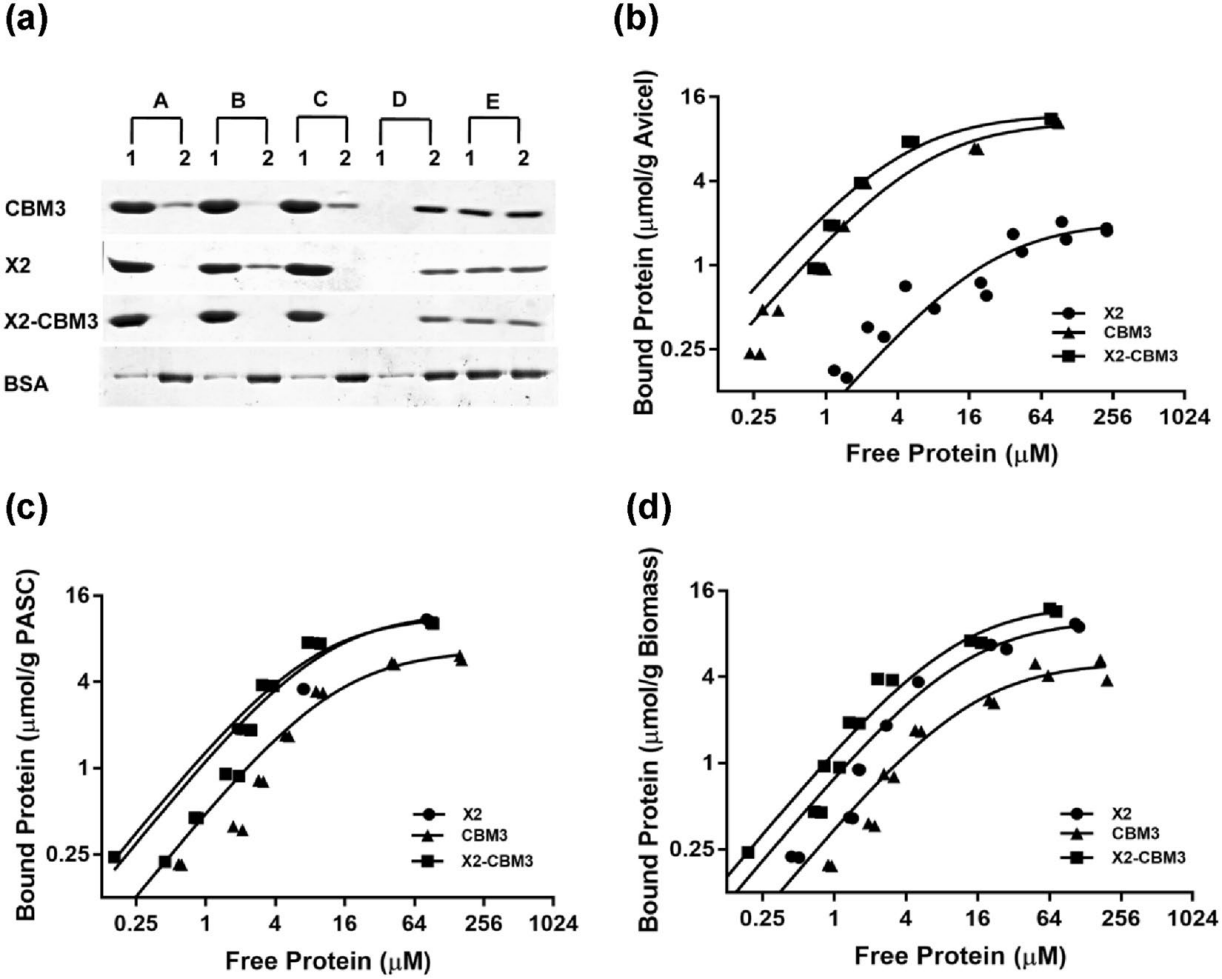
Binding of X2-CBM3 and its independent modules to insoluble polysaccharides. (**a**) 10 μg of purified protein was incubated with 4 mg of insoluble substrates, such as (A) biomass, (B) Avicel, (C) PASC, (D) starch and bound (1) and unbound* (2) proteins were analyzed on the SDS-PAGE gel. (E) The same amount of protein used in the binding assay in absence of the polysaccharide was included as a control to observe aggregation over the incubation period. Same amount of BSA was allowed to bind to the respective substrates and included as a control. Depletion isotherm of CBM3, X2 and X2-CBM3 proteins binding to (**b**) Avicel, (**c**) PASC and (**d**) biomass. X and Y axis are plotted on log2 scales for representation of data from 2-fold increasing protein concentration. Full-length gels are included as Supplementary Fig. S4. *Only 20 μl of 250 μl unbound fraction was loaded on the SDS-PAGE gel.

A depletion assay was performed to determine the affinity of proteins towards the insoluble substrate. We included biomass, Avicel, PASC and insoluble starch in our assay. BSA was used as a control for the binding assay because it does not contain any CBM. SDS-PAGE analysis of the bound and unbound fractions and dissociation constant (K_d_) measurement suggested that module X2 had a higher affinity towards biomass and PASC as compared to CBM3 (Fig. 5 and Table 3). CBM3, on the other hand, had a higher affinity towards Avicel. These data suggested that X2 might be binding to the amorphous portion of the insoluble substrate as both biomass and PASC would have significant amorphous portion due to alkaline and acid treatment, respectively. X2-CBM3 showed a higher affinity towards all the three insoluble substrates. Negligible binding was observed with the insoluble starch. BSA, being the control for the experiment, showed low non-specific binding to all the substrates. Overall, the presence of module X2 and CBM3 in association was found to enhance the binding of the polypeptide to the insoluble and crystalline substrates.

**Table 3:** Binding affinities and capacities of independent modules towards insoluble substrates as determined by binding isotherms*.

Protein	Biomass		Avicel		PASC	
	K_d_	B_max_	K_d_	B_max_	K_d_	B_max_
X2	12.12 ± 1.9	10.07 ± 0.25	24.15 ± 6.9	2.1 ± 0.22	9.96 ± 0.3	12.2 ± 0.3
CBM3	14.28 ± 3.5	5.15 ± 1.17	6.37 ± 0.07	10.6 ± 0.02	12.94 ± 1.15	6.68 ± 0.32
X2-CBM3	10.1 ± 1.15	13.02 ± 0.4	4.5 ± 0.53	12.17 ± 0.2	7.95 ± 0.3	11.57 ± 0.25
BSA	No binding		No binding		No binding	

^*^Values for K_d_ (µM) and B_max_ (µmol/g) were obtained from curve fitting using binding data presented in Fig. 5.

### Influence of X2-CBM3 on hydrolytic activity of Endo5A and Xyl11D

**Figure 6:**
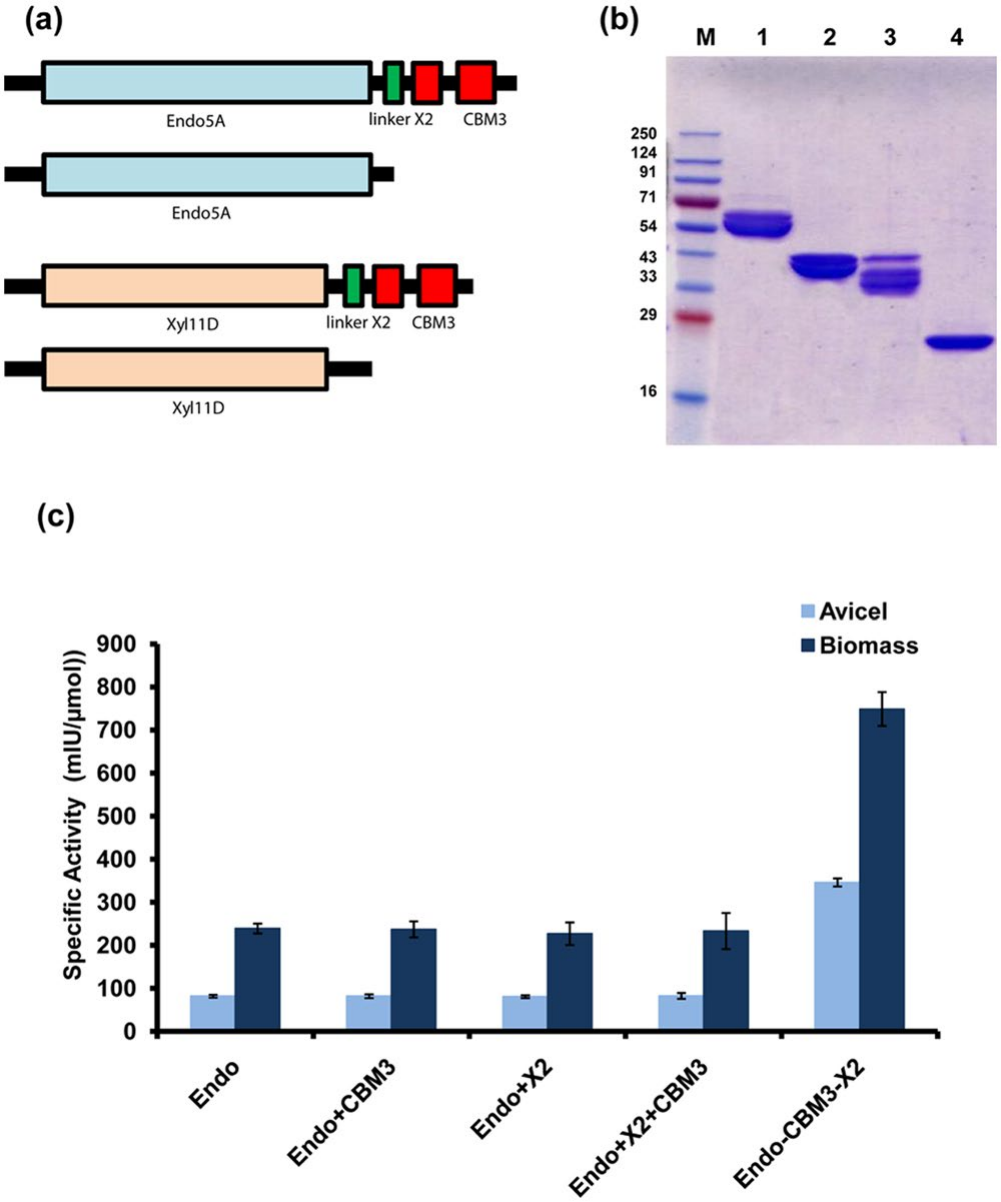
The impact of X2-CBM3 on the activity of endoglucanase (Endo5A) and xylanase (Xyl11D). (**a**) Domain organization of fusion construct containing Endo5A and Xyl11D along with X2-CBM3 linked through a Gly-Ser linker, (**b**) SDS-PAGE profile of fusion enzyme Endo5A-X2-CBM3 (Lane1), native endoglucanase Endo5A (Lane 2), fusion enzyme Xyl11D-X2-CBM3 (Lane 3), and native xylanase Xyl11D (Lane 4); M: molecular weight marker in kDa. (**c**) Enzyme activity of endoglucanase for native and fusion constructs measured towards insoluble substrates- Avicel and biomass. Enzyme activity for xylanase was not included as the activity of both of its constructs towards insoluble substrate was negligible (see text and Table 4 for details).

We observed in the previous section that X2-CBM3 influenced enzymatic activity of GH74 catalytic domain in PP3 towards the insoluble substrate. Can this carbohydrate binding domain enhance the hydrolytic potential of other glycoside hydrolases that are not associated with any CBM towards insoluble substrate? In the earlier study, we reported the characterization of an endoglucanase (Endo5A) and a xylanase (Xyl11D) from *Paenibacillus polymyxa* ICGEB2008 isolated from the gut of the cotton bollworm^42^. Both these enzymes are not associated with any CBM and have poor activity towards insoluble biomass. We thus made constructs to produce Endo5A and Xyl11D fused with module X2-CBM3 at their C-terminus and purified these fusion proteins via immobilized metal affinity chromatography (Fig. 6a and b). A glycine-serine (GS) linker was introduced between the catalytic domain and carbohydrate binding modules to avoid interference during folding. We further evaluated the activity of fusion proteins towards relevant substrates along with their native counterpart. The recombinant proteins having endoglucanase activity were analyzed for their hydrolytic potential by estimating the activity against the crystalline and insoluble substrates- Avicel and biomass, and soluble amorphous substrate- carboxymethyl cellulose (CMC). It was observed that the endoglucanase fused with X2-CBM3 demonstrated 4.2- and 3.4-fold higher hydrolysis of Avicel and biomass, respectively, as compared to native endoglucanase (Fig. 6c and Table 4); while the lesser impact was observed on the hydrolysis of amorphous substrate CMC (Table 4). A physical mixture of free CBM3, X2 or both had no impact on endoglucanase activity on either substrate (Fig. 6c). Fusion of X2-CBM3 to xylanase catalytic domain, however, did not lead to any improvement in its activity towards insoluble biomass. Also, no increase in hydrolysis of xylan was observed (Table 4). These observations suggested that while X2-CBM3 could bind to crystalline and insoluble carbohydrates, its assistance in improving the catalytic activity of a given enzyme depends solely on the substrate recognizing ability of the catalytic domain.

**Table 4:** Activities of endoglucanase Endo5A and xylanase Xyl11D fused with X2-CBM3.

Encoded protein		Substrate	Specific activity (IU/mg or mIU/mg)*	Molar specific activity (IU/μmol or mIU/μmol)*	Fold change
Endoglucanase (Endo5A)	Endo5A	CMC	22.1 (±0.6)	1014 (±17)	1
	Endo5A-X2-CBM3		26.8 (±1.0)	1480 (±31)	1.5
	Endo5A	Avicel	1.6 (±0.17)	82.24 (±3.4)	1
	Endo5A-X2-CBM3		5.43 (±0.2)	346.32 (±9.2)	4.2
	Endo5A	Ammonium-treated biomass	1.17 (±1.1)	239.2 (±11)	1
	Endo5A-X2-CBM3		3.72 (±1.2)	749 (±39)	3.4
Xylanase (Xyl11D)	Xyl11D	Xylan	76.5 (±3.9)	1706 (±88)	1
	Xyl11D-X2-CBM3		47.8 (±3.5)	2025 (±150)	1.2
	Xyl11D	Ammonium-treated biomass	0.0 (±0.0)	0.0 (±0.0)	NA
	Xyl11D-X2-CBM3		0.0 (±0.0)	0.0 (±0.0)	NA

*Units in IU/mg or IU/μmol are given for amorphous substrates CMC and xylan. Units in mIU/mg or mIU/μmol are given for crystalline substrates Avicel and ammonium-treated biomass.

### Phylogenetic analysis and structural modeling of module X2 of *P*. *polymyxa* A18

**Figure 7:**
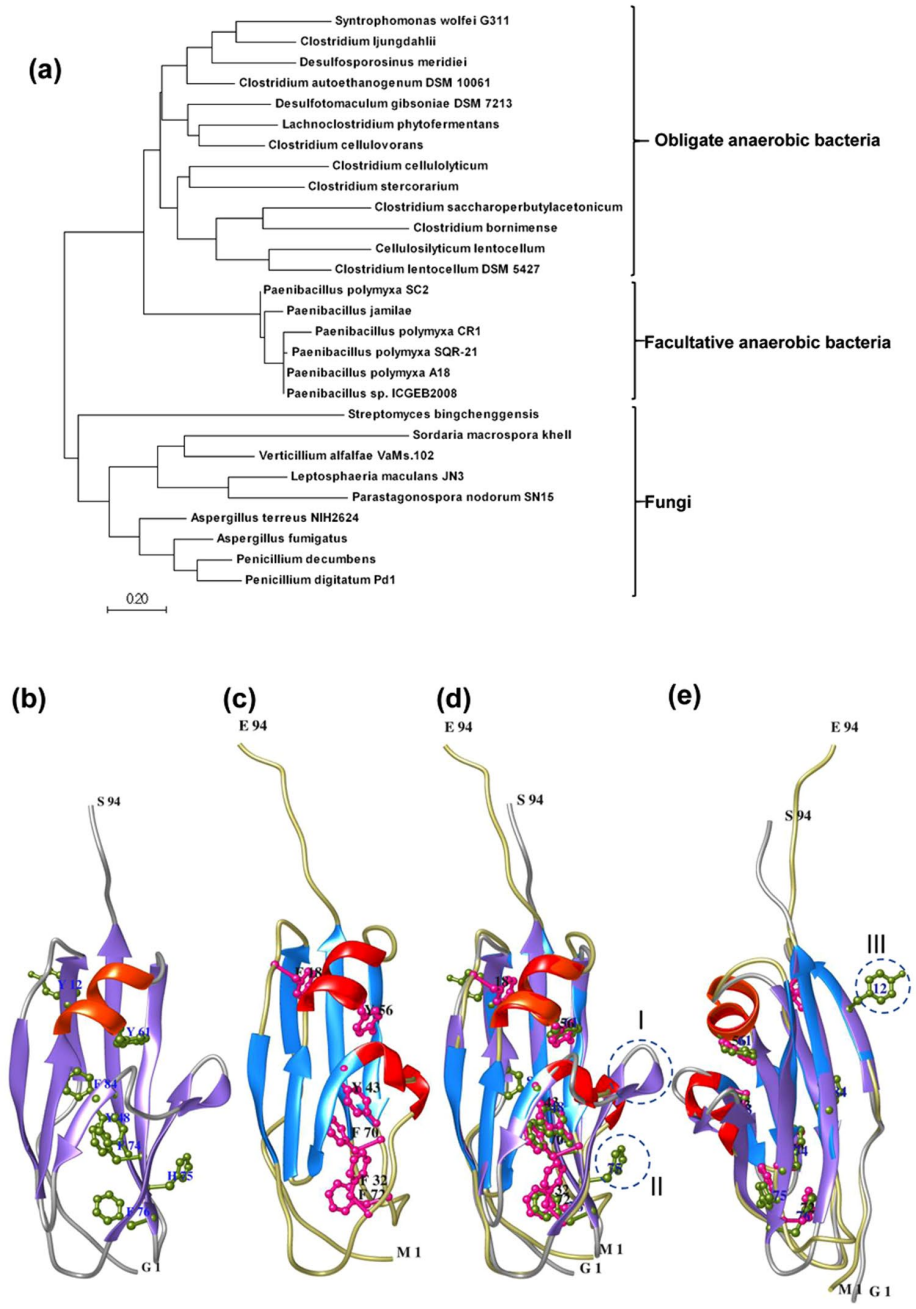
*In silico* analysis of module X2 of PP3 of *P*. *polymyxa* A18. (**a**) Molecular Phylogenetic analysis was performed by Maximum Likelihood method and the tree with the highest log likelihood is shown. Modelled structure of PP3-X2 (**b**), NMR-based structure of CipC-X2 (**c**) and superimposition of the two structures in two orientations (**d**,**e**) have been shown.

Module X2 played a significant role in substrate recognition and its association with CBM3 was found to be useful for the associated enzymes to perform their catalytic activity. We thus analyzed the amino-acid sequence of PP3-X2 for various primary, secondary and tertiary features. The region X2 in PP3 (790 to 858 amino acid, Fig. 2) and related sequences listed in NCBI database were used for phylogenetic analysis by Maximum Likelihood method. The analysis grouped X2 encoded by various organisms into three clades, those encoded by (1) obligate anaerobic bacteria, (2) facultative anaerobic bacteria, and (3) fungi (Fig. 7a). As expected, the X2 from *P*. *polymyxa* A18 segregated in facultative anaerobic bacteria clad. The multiple sequence alignment highlighted the X2 related conserved sequence ‘NGNT’ towards the N-terminus of X2 of PP3 (Supplementary Fig. S5), as was noted earlier^43^.

We performed structural modeling of PP3-X2 with available structures of module X2 in Protein Data Bank (PDB). Amongst the two structures available for module X2 in PDB, PP3 X2 showed higher structural homology with X2 of CipC protein of *Clostridium cellulolyticum* as compared to CBM4–2 X2-L110F of *Rhodothermus marinus* (Supplementary Fig. S6). The superimposition of modelled structure of PP3-X2 with CipC-X2 structure has been described in Fig. 7(b–e). The overall folds in these two structures were very similar to one another (Fig. 7b and c). As expected, immunoglobulin-like fold composed of two β-sheets was present. The major differences, however, have been highlighted in circles in the superimposed model in Fig. 7d and e. Circle I represents a change in one of the two α-helices in CipC-X2 into a β-turn in PP3-X2. Circle II and Circle III represent a histidine and an aromatic amino acid phenylalanine, respectively, in PP3-X2 protruding outside, as against CipC-X2 where histidine is replaced with aspartate (Supplementary Fig. S6a) and all aromatic amino acids are clustered towards core (Fig. 7c). These differences might have implications towards binding of PP3-X2 to the carbohydrate polymers.

## Discussion

For commercial purposes, biofuel production from lignocellulosic biomass has been a major target for developing biotechnological conversion processes. Despite recent developments, saccharification of lignocellulose is still a key process which requires technical and economic improvements. The conversion of biomass to platform sugars is hindered primarily by the complexity of lignocellulosic substrates as well as by the performance of the available enzymes^44^. The inherent challenge lies in the recalcitrance of plant cell walls comprising of a highly heterogeneous and stable structure of cellulose, hemicellulose, and lignin^3^. Nature has provided especial arm in the form of CBMs to some of the carbohydrate active enzymes to deal with this challenge by enhancing the ability of the enzyme to recognize and bind to the carbohydrate substrate. The aim of our current study has been to identify the CBMs produced by the cellulolytic microbe *P*. *polymyxa* A18, which was isolated from the termite gut, and assess them in terms of their substrate binding properties and their ability to enhance the catalytic activity of associated enzymes. Our previous study showed that *P*. *polymyxa* A18 has the ability to grow on alkali-treated biomass by producing enzymes that can efficiently hydrolyse this biomass^22^.

We first annotated the genome of *P*. *polymyxa* A18 for the presence of CBMs encoding open reading frames. Since variations were observed in the annotation results of Carbohydrate active enzyme ANnotation (dbCAN) and CAZyme Analysis Toolkit (CAT), we proceeded with the consensus domains obtained from the analysis of the annotation results. 19 CBMs were found to be present in 14 polypeptides. We found that CBM3 dominated amongst all the CBMs and was present in 4 polypeptides containing endoglucanase (GH5), cellobiohydrolase (GH6), xyloglucanase (GH74) and arabinofuranosidase/mannanase (GH44/GH26) activity. Several studies earlier showed affinity of CBM3 towards crystalline substrate and excision of this module from the polypeptide led to a decline in activity of the associated enzymes towards crystalline substrate^17, 45, 46^. In this context, CBM3 containing polypeptides encoded by A18 genome seemed to play a crucial role in biomass degradation. This was further confirmed by qRT-PCR that was performed to monitor the expression of genes encoding polypeptides containing CBMs. We observed a high level of expression of genes encoding CBM3 along with the catalytic modules of cellobiohydrolase (PP2) and xyloglucanase (PP3) in response to insoluble biomass and crystalline cellulose. But the most remarkable increase observed in the expression that caught our attention was a 30–40-fold increase in the expression of PP3 in response to these insoluble substrates (Fig. 3a and b). Previous reports also suggested that the expression of enzymes and associated CBMs responsible for digestion of insoluble and crystalline carbohydrates is tightly controlled, and are highly inducible in nature depending upon the kind of substrate present in the growth medium^30, 31, 47^.

A closer look at the architecture of PP3 indicated that it consists of a catalytic module of xyloglucanase of the GH74 family followed by module X2 and CBM3 (Fig. 2). A similar polypeptide named XEG74 from *Paenibacillus* sp. strain KM21 has been recombinantly expressed earlier and shown to have both exo- and endo-type activity or processive endo-type activity on xyloglucan^35^. However, neither the role of module X2-CBM3 on xyloglucanase activity nor the activity of xyloglucanase on the insoluble substrate has been deciphered. The presence of module X2 in this polypeptide along with CBM3 structurally resembles that of cellulosomal scaffoldin protein CipC of *Clostridium cellulolyticum*, except that CipC has multiple cohesins to hold dockerin module present in the enzyme rather than having a catalytic module of its own^43^. Solution structure of module X2 present in CipC suggested that it has immunoglobulin-like conformation and might be involved in stability and/or solubility of scaffolding protein^43^. It was also predicted that module X2 helps in cellulosome attachment on the cell surface^48^, although direct binding between module X2 and any component of the cell surface of *C*. *cellulolyticum* could not be demonstrated. Moreover, no information is available on the role of module X2 present in the enzymes of mesophilic bacteria.

We, therefore, expressed full- length and truncated forms of PP3 such that the impact of module X2 and CBM3 could be determined on the catalytic activity of the associated xyloglucanase. These modules were found to have a significant impact on the xyloglucanase activity towards soluble and insoluble substrates. The presence of individual modules also improved the enzyme activity, but together they were found to have a substantial impact. These results suggest that module X2 might also be assisting in binding to the insoluble substrate along with CBM3, thus contributing to enzyme hydrolysis.

To address the substrate binding properties of module X2 and CBM3, these modules were recombinantly expressed as independent modules as well as in combination and were assessed for the binding towards soluble xyloglucan and insoluble pre-treated biomass, PASC, and Avicel. Considerable binding interactions were observed for CBM3 and X2-CBM3 with soluble xyloglucan in ITC experiments (Supplementary Fig. S3 and Table 2). The negative enthalpy change in CBM3 suggested favorable interactions with xyloglucan which resulted into significant binding affinity, however, the negative contribution from entropy is perhaps because of the reduced molecular freedom of binding partners upon complex formation^49^. The binding of X2-CBM3 resulted in lower affinity with interactions driven mainly by positive entropy (Table 2) and unfavorable enthalpy. Entropy-driven interactions usually result from the release of water molecules from the binding interface of ligand and peptide upon complex formation suggesting the contribution of desolvation entropy in the favorable interaction^49, 50^. The unfavorable enthalpy change suggested a net loss of non-covalent interactions in complex formation^49, 50^. Binding was also observed between X2 and xyloglucan, though the exact parameters could not be calculated due to the complexity of the curve obtained.

All modules showed significant binding towards Avicel, PASC, and biomass in substrate binding studies, but not with insoluble starch, suggesting that they were directed towards the carbohydrate polymer of biomass (Fig. 5a). CBM3 has been shown earlier to bind strongly with the crystalline substrate^35, 45^; we also found high affinity of CBM3 towards Avicel (Fig. 5b). Module X2 had relative weak binding with Avicel. However, when Avicel was treated with phosphoric acid (PASC) to make its surface amorphous, the binding of X2 increased significantly with the corresponding decrease in binding of CBM3 (Fig. 5c). Module X2 also showed higher binding affinity to pre-treated biomass (Fig. 5d), which is likely to have amorphous regions on the surface too. Interestingly, the presence of X2 along with CBM3 increased its binding affinity to both PASC and biomass, thus highlighting the role of X2 in improving the flexibility and robustness of the associated enzymes for binding to their substrates.

Pre-treated biomass is insoluble but highly heterogenous in nature and thus requires a variety of enzymes and accessory proteins for its digestion. X2-CBM3 seems to have high affinity towards the pre-treated biomass. It would be interesting to see if it can also contribute to the activity of other enzymes that are required for digestion of biomass and do not contain CBMs inherently. When module X2-CBM3 was fused with an endoglucanase, Endo5A, the activity of the enzyme increased by 4.2-fold on the insoluble substrate; while the impact was minimal with respect to the soluble substrate. Fusion with xylanase, however, did not lead to any increase in its activity with either soluble or insoluble substrate. The positive impact of module X2-CBM3 on Endo5A could be because like xyloglucanase this endoglucanase is also processive in nature as it releases cellobiose as the product^42, 51^. Several reports in literature manifest that catalytic activity of cellulase/hemicellulose upon fusion with CBMs^52–56^ gets improved, but it is the first comprehensive report which emphasizes that the activity of glycoside hydrolases gets significantly improved by the combination of module X2 and CBM3.

Our results showed the ability of module X2 of PP3 of *P*. *polymyxa* A18 to bind to carbohydrate polymers; this feature made it distinct from earlier known module X2 of CipC of *C*. *cellulolyticum* ^43^. The phylogenetic analysis revealed module X2 of *P*. *polymyxa* A18 forming a separate clad along with those from other mesophylic species as compared to module X2 of *C*. *cellulolyticum* (Fig. 7a). The solution structure of module X2 of *C*. *cellulolyticum* revealed immunoglobulin-like domain with all the aromatic residues buried inside^43^ (Supplementary Fig. S7b). This characteristic was opposite to that of the typical carbohydrate binding modules (CBMs) where the majority of the aromatic residues have been shown to protrude outside in the solvent exposed area to help in ligand binding^40^ (Supplementary Fig. S7c) and thus was believed not to bind to carbohydrate polymer. However, the presence of an aromatic residue (Circle III in Fig. 7e) on the surface and an additional β-strand (Circle I in Fig. 7d) suggested that PP3-X2 might be contributing to ligand binding. A histidine residue conserved in *Paenibacillus* species (position 69 in Supplementary Fig. S5) was also found on the surface (Circle II in Fig. 7d and Supplementary Fig. S7a), which had been suggested earlier to contribute towards ligand binding in other carbohydrate module^57, 58^. These structural features may provide the basis for the robust binding of module X2 observed on the amorphous surface of the insoluble substrate.

To conclude, our study identified a carbohydrate binding polypeptide X2-CBM3 that was maximally expressed by *P*. *polymyxa* A18 in response to biomass. Further characterization proved its ability to assist in biomass and crystalline cellulose hydrolysis. X2 and CBM3 together were demonstrated to be highly efficient in enhancing the activity of glycoside hydrolases, such as xyloglucanase of GH74 family and endoglucanase of GH5 family, towards the insoluble substrate. This novel CBM combination will allow designing of cellulases and other enzymes for improved biomass hydrolytic potential and hence lead to low-cost biofuel production.

## Methods

### Identification of CBMs in *Paenibacillus polymyxa* A18 genome

*P*. *polymyxa* A18, a cellulolytic bacterium, was isolated from the termite gut in a previous study^22^. Its genome sequence is available at DDBJ/EMBL/GenBank under accession number JWJJ00000000. RAST server (http://rast.nmpdr.org/) was used to predict putative proteins in the genome^59, 60^. Carbohydrate Binding Modules (CBMs) were screened in the genome using the programs available for prediction of Carbohydrate-active modules. CAZyme analysis toolkit^24^ (CAT) predicts modules on the basis of sequence similarity as well as on the links it has generated between Protein families (Pfam) and CAZy families. Carbohydrate-active enzyme ANnotation^23^ (dbCAN) has a set of HMM profiles for each CAZy class on the basis of which it predicts the modules. CAT (v2.0) was used at default parameters for annotation of CBMs whereas dbCAN release 3.0 was used at an E-value cut-off of 10^−16^ (GH), 10^−5^ (GT), 10^−11^ (PL), 10^−16^ (CE), 10^−12^ (CBM) and 10^−11^ (AA) to predict various CAZymes. These cut-off values have given a similar number of CAZy family proteins for other *P*. *polymyxa*, such as SC2 and M1 which are already present in the CAZy database.

CBMs identified were compared and only those predicted by both CAT and dbCAN were used for further analysis. Domain architectures of the proteins predicted to contain CBMs were drawn using the information obtained from HMM scan data of dbCAN. Classical signal peptides for secretion (SPs) were identified using SignalP 4.1^61^.

### Growth conditions and enzymatic activity

The seed culture for *P*. *polymyxa* A18 was grown in Tryptone Soy Broth (Himedia) at 37 °C overnight. It was then sub-cultured in mineral salts medium (6.8 g/l KH_2_PO_4_, 3 g/l NH_4_Cl, 1 g/l KCl, 0.5 g/l sodium citrate, 0.2 g/l MgSO_4_, 30 mg/l MnSO_4_, 30 mg/l EDTA, 10 mg/l CaCl_2_, 5 mg/l Na_2_MoO_4_, 5 mg/l FeSO_4_.7H_2_O, 5 mg/l H_3_BO_3_, 3 mg/l CoCl_2_.6H_2_O, 1 mg/l CuSO_4_.5H_2_O, 1 mg/l ZnSO_4_.7H_2_O, 1 mg/l nicotinic acid, 2 mg/l biotin, 2 mg/l para-aminobenzoic acid, and 2 mg/l thiamine hydrochloride) with 2 g/l tryptone and either of the carbon substrate (10 g/l) such as pretreated wheat straw, Carboxy Methyl Cellulose (CMC) low viscosity (Himedia), Avicel PH101 (Sigma), beechwood xylan (Himedia). Wheat straw used as a substrate for the growth was subjected to ammonium hydroxide pretreatment (kindly provided by Prof. Arvind Lali)^62^. The pretreated straw was graded through a 0.5 mm mesh and stored at 4 °C. Compositional analysis of this pretreated wheat straw showed that it contains approximately 68% glucose, 11% xylose, 2% arabinose, 15% lignin, and 3% ash. Phosphoric Acid Swollen Cellulose (PASC) was prepared from Avicel as described earlier^63^.

Growth was monitored directly by measuring OD_600._ To assess the cellulolytic activity, endoglucanase (β-1,4-endoglucanase) and xylanase (β-1,4-endoxylanase) activities were measured at 4-hr intervals using CMC and xylan as substrates, respectively. The reducing sugar released upon the hydrolysis of sugar polymers was quantified by dinitrosalicylic acid reagent at 540 nm using DNSA method^64^. One unit of enzymatic activity was defined as the amount of enzyme that released 1 μmol of reducing sugar from the substrate per minute under the above-mentioned conditions.

### RNA isolation and cDNA synthesis

*P*. *polymyxa* A18 was grown in 50 ml of mineral salts media as stated above with either of the carbon substrate (10 g/l). Cells grown without any carbon substrate were used as a control for the study. Cells were harvested after 8 hr of growth and stored in RNAprotect® Bacteria Reagent (Qiagen) at −80 °C until RNA extraction. Total RNA was extracted using the Qiagen RNeasy Mini Kit with on-column treatment with RNase-Free DNase (Qiagen). After ensuring the integrity of the RNA on agarose gel and quality by measuring the 260/280 nm ratio, equal amounts were converted to cDNA using the First Strand cDNA Synthesis Kit (Invitrogen) with random hexamer primers. To check for genomic DNA contamination in the cDNA, primer sets based on non-coding regions NC1 and NC2 were used (Supplementary Table S4). Samples were further processed when no contamination was found.

### Quantitative reverse transcription PCR

For gene-specific qRT-PCR, primer pairs were designed with an amplicon size of 222 bp (Supplementary Table S4). The 16S rRNA housekeeping gene was used as a reference gene. Each 20-μl qRT-PCR mixture contained 10 μl of 2× Light-Cycler 480 SYBR green mastermix, 1 μl of cDNA, and 1 μl each of 5 μM forward and reverse primers. Real-time PCR was performed on a Light Cycler 480 instrument (Applied Biosystems) according to the manufacturer’s instructions. Cycling conditions were as follows: initial denaturation at 95 °C for 4 min and 45 cycles of 95 °C for 15 s, annealing temperature as mentioned in Supplementary Table S4 for 15 s, and extension at 72 °C for 20 s. The data were interpreted using Applied Biosystems software. All assays were carried out in 96-well plates that were covered with optical tape. All the samples were analyzed in technical duplicates for two biological replicates of the experiment.

Fold change was calculated using the formula 2^−ΔΔC^_T_^37^. For a given carbon substrate, ∆C_T_ was obtained by subtracting C_T_ value observed for reference gene from the test gene. ∆∆C_T_ was then calculated as the difference between ∆C_T_ obtained in a given carbon substrate and ∆C_T_ obtained in the absence of a carbon substrate. Statistical analysis was performed using an independent Student’s *t*-test. The significance of the ∆∆C_T_ values obtained was calculated using a two-tailed unpaired Student’s *t*-test on the mean of ΔC_T_ values for a given carbon substrate compared to ΔC_T_ values obtained in its absence. P values of 0.05 and less were considered significant.

### Cloning of CAZy family proteins

Genomic DNA of *P*. *polymyxa* A18 was used as a template for amplification of all genes unless mentioned. Sequences of the primers used in PCR have been mentioned in Supplementary Table S5. Gene encoding full-length xyloglucanase was PCR amplified without the native signal sequence using a primer set Xylg_F and Xylg_R and cloned into the pQE-30 vector at *Sph*I and *Sal*I restriction sites to obtain pQE-PP3-1. pQE-PP3-3 construct encoding GH74-CBM3 was prepared by first cloning gene encoding GH74 at C-terminal in pQE30 using primer set Xylg_F/GH74_R_kpnI. CBM3 was attached to C-terminus of GH74 using primer set CBM3_F_kpnI/CBM3_R_salI. The genes encoding GH74, GH74-X2, X2-CBM3, X2 and CBM3 proteins were amplified using primer sets Xylg_F/GH74_R, Xylg_F/X2_R_salI, X2_F_sacI/CBM3_R_salI, X2_F_sacI/X2_R_salI and CBM3_F_sacI/CBM3_R_salI and cloned into pQE30 at *Sac*I and *Sal*I restriction sites to obtain pQE-PP3-4, pQE-PP3-2, pQE-X2-CBM3, pQE-X2 and pQE-CBM3 constructs, respectively.

The X2-CBM3 stretch was appended at the C-terminus of Endo5A and Xyl11D as follows. Constructs from a previous study^42^, i.e., pQE-Endo5A-GS-Xyl11D and pQE-Xyl11D-GS-Endo5A, were modified by introducing X2-CBM3 amplicon downstream of the GS linker at *Sac*I and *Sal*I restriction sites to obtain pQE-Endo5A-GS-CBM and pQE–Xyl11D-GS-CBM plasmid, respectively. All constructs were designed to express proteins along with a 6-histidine tag to facilitate affinity purification.

### Protein expression and purification

The *E*. *coli* DH5α strain transformed with plasmid constructs was used for the expression and purification of recombinant xyloglucanase, GH74, CBM3, X2, X2-CBM3, GH74-CBM3, GH74-X2, Endo5A-GS-CBM, and Xyl11D-GS-CBM. Cells were grown in LB medium (500 ml) containing ampicillin (100 μg/ml) and induced with 0.1 mM IPTG (isopropyl-β-D-thiogalactopyranoside) at an OD_600_ of 0.6. The cultures were then grown for an additional 4 h and harvested by centrifugation. The cells were lysed by sonication in lysis buffer (50 mM NaH_2_PO_4_, 300 mM NaCl, 10 mM imidazole pH 7.4) and clarified by centrifugation. The resultant supernatant was used to purify the recombinant protein using Immobilized Metal Affinity Chromatography (IMAC). The clarified cell lysate was loaded onto a Ni-Nitrilo Triacetic Acid (Ni-NTA) agarose matrix, washed with buffer containing 20 mM imidazole and eluted with buffer containing 250 mM imidazole. The eluted enzymes were buffer exchanged with 50 mM sodium phosphate (pH 7) using a 10 kDa cut-off filter (Millipore) and used for purity determination by SDS- PAGE. The protein concentrations were determined using the bicinchoninic acid assay (BCA) reagent kit (GE Healthcare) with bovine serum albumin as the standard.

### Interaction of X2, CBM3, and X2-CBM3 with Insoluble Polysaccharides

Binding of X2, CBM3, and X2-CBM3 to the insoluble polysaccharides was determined by the following method. Equal quantities of purified proteins (10 μg) were mixed with insoluble polysaccharides- pretreated biomass, Avicel, PASC and starch (4 mg) in 10 mM sodium phosphate buffer (pH 6.3) to make up the final volume of 250 µl. Entire mixture was incubated on ice for 12 h with occasional stirring. After centrifugation, supernatants were collected (unbound protein) and the pellets were washed twice with 10 mM sodium phosphate buffer (pH 7). Pellets were then treated with SDS-PAGE dye, which along with 20 µl of supernatant was analyzed on 15% SDS-PAGE gel for the presence of proteins. BSA was used as a negative control. Controls with proteins but no ligands were included to ensure that precipitation did not occur during the assay. Colloidal Blue (G biosciences) was used to stain the gels.

Depletion isotherms to quantify the binding of X2, CBM3, and X2-CBM3 to insoluble substrates Avicel, PASC, and biomass were carried out by mixing 1–256 μM protein with 10 mM sodium phosphate buffer (pH 7) containing 4 mg of each substrate. The mixture was incubated at 4 °C for 12 h. Samples were centrifuged at 10,000 g, 4 °C for 2 min to pellet the bound substrate, and unbound proteins in the supernatant were quantified using the Pierce BCA protein assay kit using the formula: bound protein = total protein − unbound protein. Dissociation constant (K_d_) and the amount of protein bound at saturation (B_max_) were calculated by fitting the data to a single site Langmuir isotherm using GraphPad Prism 7.02 (GraphPad Software, Inc., San Diego, CA). Two separate binding isotherms were carried out for each protein.

### Isothermal titration calorimetry

All ITC experiments were carried out at 298 K on a Microcal iTC_200_ (GE healthcare) calorimeter in 10 mM sodium phosphate buffer pH 7. For each experiment, protein concentration (X2, CBM3 and X2-CBM3) of 50 μM and 1.5% w/v polysaccharide concentration (xyloglucan - Megazyme) was used. Ligand was subjected as 1^st^ injection of 0.4 μl with an initial delay of 60 s followed by 19 injections of 2 μl each at 150 s intervals. Since the molar concentration of the complex polysaccharide could not be determined, the molar concentration of binding sites (n) on the polysaccharides was set to the value of 1 with the help of Origin software during the analysis. This approach assumes that proteins have only one binding site^41^. The heat of dilution was monitored by injecting the ligand into the buffer. These reference values were subtracted from the integrated data prior to fitting. Integrated heat effects were analyzed by non-linear regression using a single-site binding model. Origin 7 software was used for the fitting of nonlinear regression using a single-site model. The affinity constant (K_A_) and the enthalpy of binding (ΔH) were obtained by the integration. Other thermodynamic parameters were calculated by using the following equation:

$$-{\rm{RT}}\,\mathrm{ln}\,{{\rm{K}}}_{{\rm{A}}}={\rm{\Delta }}{\rm{G}}={\rm{\Delta }}{\rm{H}}-{\rm{T}}{\rm{\Delta }}{\rm{S}}.$$

Thermodynamic parameters and affinity constant were calculated only after subtracting the heat observed from the control experiment such that substrate-only heat does not have any influence on the calculated parameters.

### Characterization of recombinant xyloglucanase and its truncated derivatives

Enzymatic activity was measured on soluble polysaccharide substrates, i.e, carboxymethyl cellulose (CMC), xylan (beechwood) and xyloglucan (tamarind), and insoluble polysaccharides, i.e., Avicel and ammonium-treated wheat straw (biomass) using the dinitrosalicylic acid (DNSA)-based reducing sugar determination assay. Enzyme solution (equal moles of proteins were taken for comparison) was mixed with 0.125 ml of 1% substrate solution in 0.1 M sodium phosphate buffer (pH 7.0) and incubated at 50 °C for 30 min for soluble substrates and 4 h for insoluble substrates. The reducing sugar produced in these experiments was measured with the DNSA reagent at 540 nm as mentioned earlier^22^. One unit of enzyme activity was defined as the amount of enzyme required to release 1 μmol of reducing sugars from the substrate per minute under the above-mentioned conditions.

Specificity of recombinant xyloglucanase and its truncated derivatives were compared by the pattern of cleavage of all its four variants. The digestion products of tamarind xyloglucan were analyzed using HPLC and mass spectrometry. Xyloglucan was incubated at 50 °C for 18 h in 10 mM sodium phosphate buffer (pH 7.0) containing the recombinant enzyme. The final products were analyzed by normal-phase HPLC-RID and mass spectrometry. HPLC was carried out using a Hi-Plex Na column (Agilent) using 100% water (isocratic) at a flow rate of 0.3 ml/min. HPLC fractions were collected and analyzed using 3200 QTRAP (AB Sciex) mass spectrometer at ion spray voltage of 5500 V in positive mode -ion detection.

### Phylogenetic analysis of module X2

Amino acid sequences of module X2 were retrieved from GenBank (http://www.ncbi.nlm.nih.gov/) database. Alignment was performed with Clustal Omega^65^ and Jalview^66^ tool was used to visualize the alignment file. The phylogenetic tree was constructed using the Neighborjoining method implemented with the MEGA version 7 program^67^, based on the alignment produced with Clustal Omega.

### Model construction

Three-dimensional (3-D) structure of module X2 was developed by homology modeling method. The molecular model was generated by MODELLER^68^ taking multiple templates having Protein Data Bank (PDB) accession number 1EHX, 4UZN and 4V2X. Chimera^69^ was used to visualize the structural models. Secondary structural features presented in the alignment were depicted using ESPript^70^.

## Electronic supplementary material

Supplementary Figures and Tables
