# Supplementary material for: Impact of Module-X2 and Carbohydrate Binding Module-3 on the catalytic activity of associated glycoside hydrolases towards plant biomass
Source: Sci Rep. 2017 Jun 16;7:3700. doi: 10.1038/s41598-017-03927-y (PMC5473887; doi:10.1038/s41598-017-03927-y)
Supplement: Supplementary file 1 — Supplementary Figures and Tables [file 41598_2017_3927_MOESM1_ESM.pdf]

Supplementary Information

Impact of Module-X2 and Carbohydrate Binding Module-3 on the catalytic activity of associated glycoside hydrolases towards plant biomass

Nandita Pasari[1], Nidhi Adlakha[4], Mayank Gupta[1], Zeenat Bashir[1], Girish H Rajacharya[2], Garima Verma[3], Manoj Munde[5], Rakesh Bhatnagar[4] and Syed Shams Yazdani[1,2*]

Supplementary information provided with this submission:

Figure S1. Growth and cellulolytic activity of *P. polymyxa* A18.

Figure S2. HPLC (left column) and mass spectrometry (right column) profiles of the enzymatic hydrolysate of tamarind xyloglucan after incubation with full-length and truncated forms of xyloglucanase.

Figure S3. Isothermal titration calorimetry (ITC) data for binding interactions with soluble xyloglucan of (a) CBM3, (b) X2-CBM3, (c) X2, and (d) buffer (control) **.**

Figure S4. Full-length SDS-PAGE gels for binding assay of X2-CBM3 and its independent modules to insoluble polysaccharides.

Figure S5. Alignment of module X2 of different organisms.

Figure S6. Alignment of X2 of PP3 of *P. polymyxa* A18 with (a) X2 of CipC of *Clostridium cellulolyticum* and (b) CBM4-2 X2-L110F of *Rhodothermus marinus*.

Figure S7. (a) Structural model of *P. polymyxa* A18 PP3-X2 as predicted using MODELLER. Representations of already known structures (b) of CipC-X2 of *C. cellulolyticum* (PDB ID 1EHX) and (c) of CBM46 of *Bacillus halodurans* (PDB ID 4UZ8).

Table S1. Annotation of Carbohydrate Binding Modules (CBMs) in *P.polymyxa* A18 genome.

Table S2. Relative transcript levels of genes encoding for CBM containing polypeptides in the presence of different carbon substrates.

Table S3. Comparison of xyloglucanase activity of truncated derivatives towards soluble and insoluble substrates.

Table S4. List of primers used for qRT-PCR.

Table S5. Strains, plasmids, and primers used in the study.

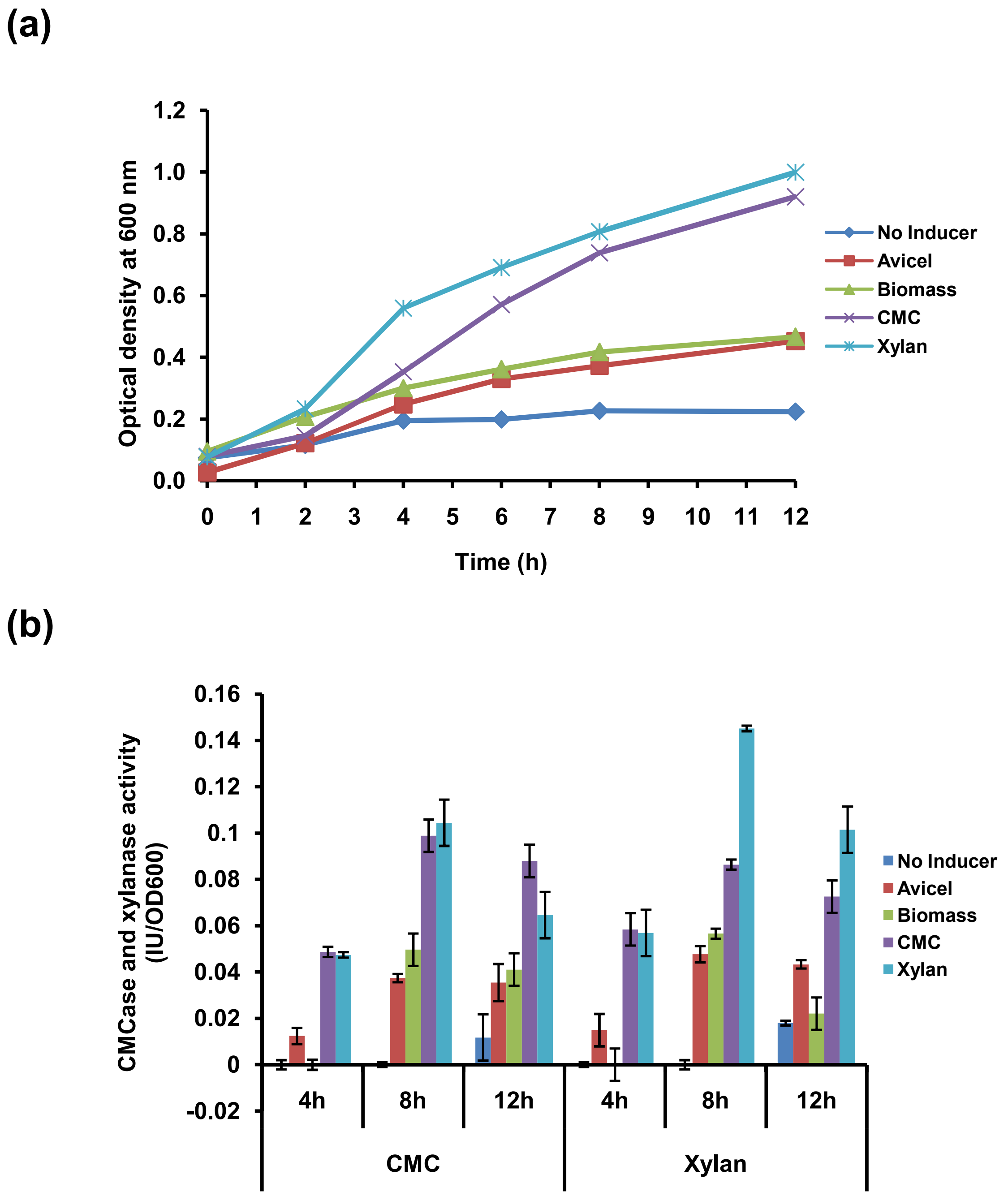

Figure S1. Growth and cellulolytic activity of *P. polymyxa* A18. (a) *P.polymyxa* A18 was grown in different carbon substrates and its growth was monitored by measuring OD_{600} at an interval of 2 hr up to 12 hr. (b) Hydrolytic activity was monitored in the supernatant of *P. polymyxa* A18 grown in different carbon substrates by measuring CMCase and Xylanase activity at an interval of 4 hr.

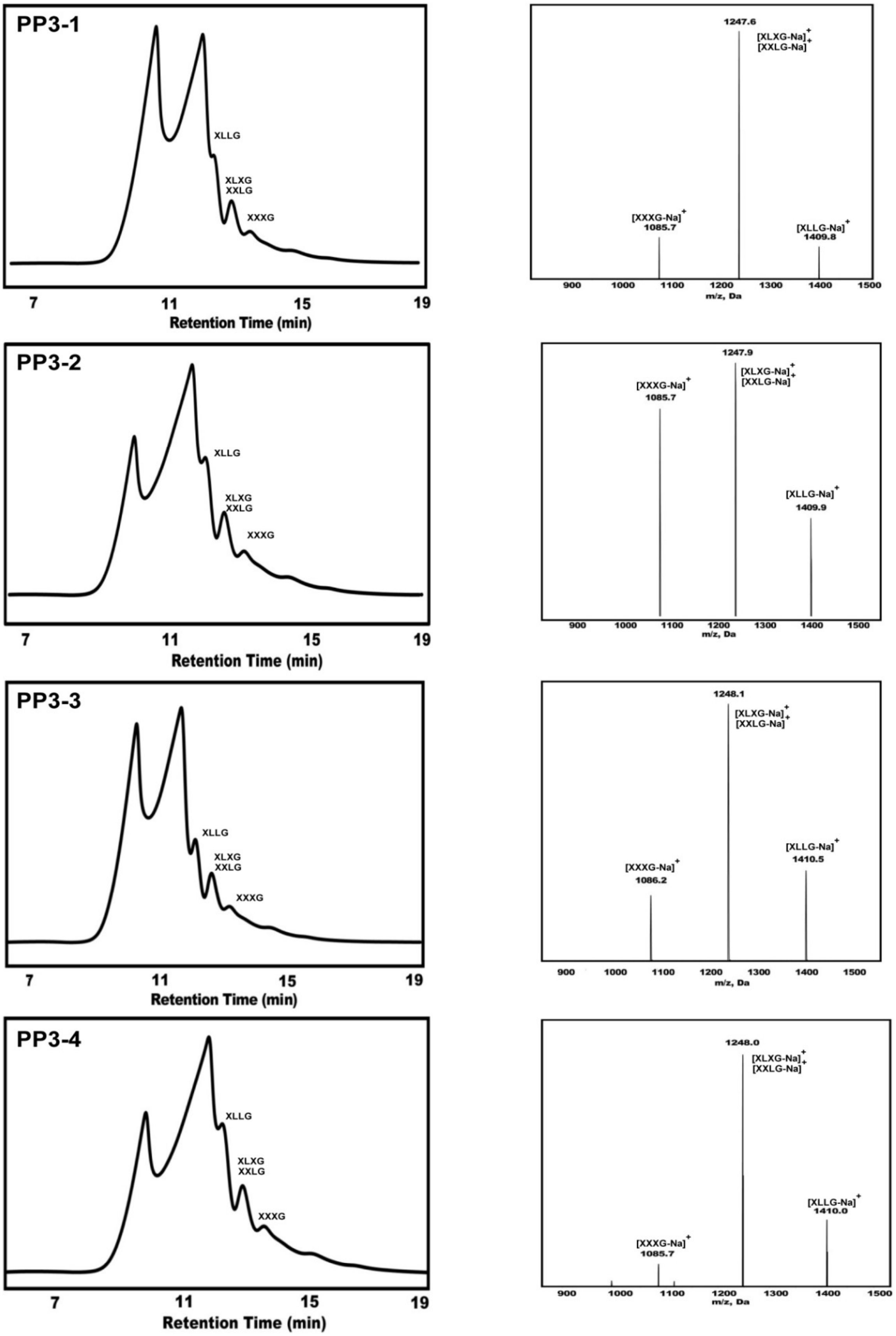

Figure S2. HPLC (left column) and mass spectrometry (right column) profiles of the enzymatic hydrolysate of tamarind xyloglucan after incubation with full-length and truncated forms of xyloglucanase.

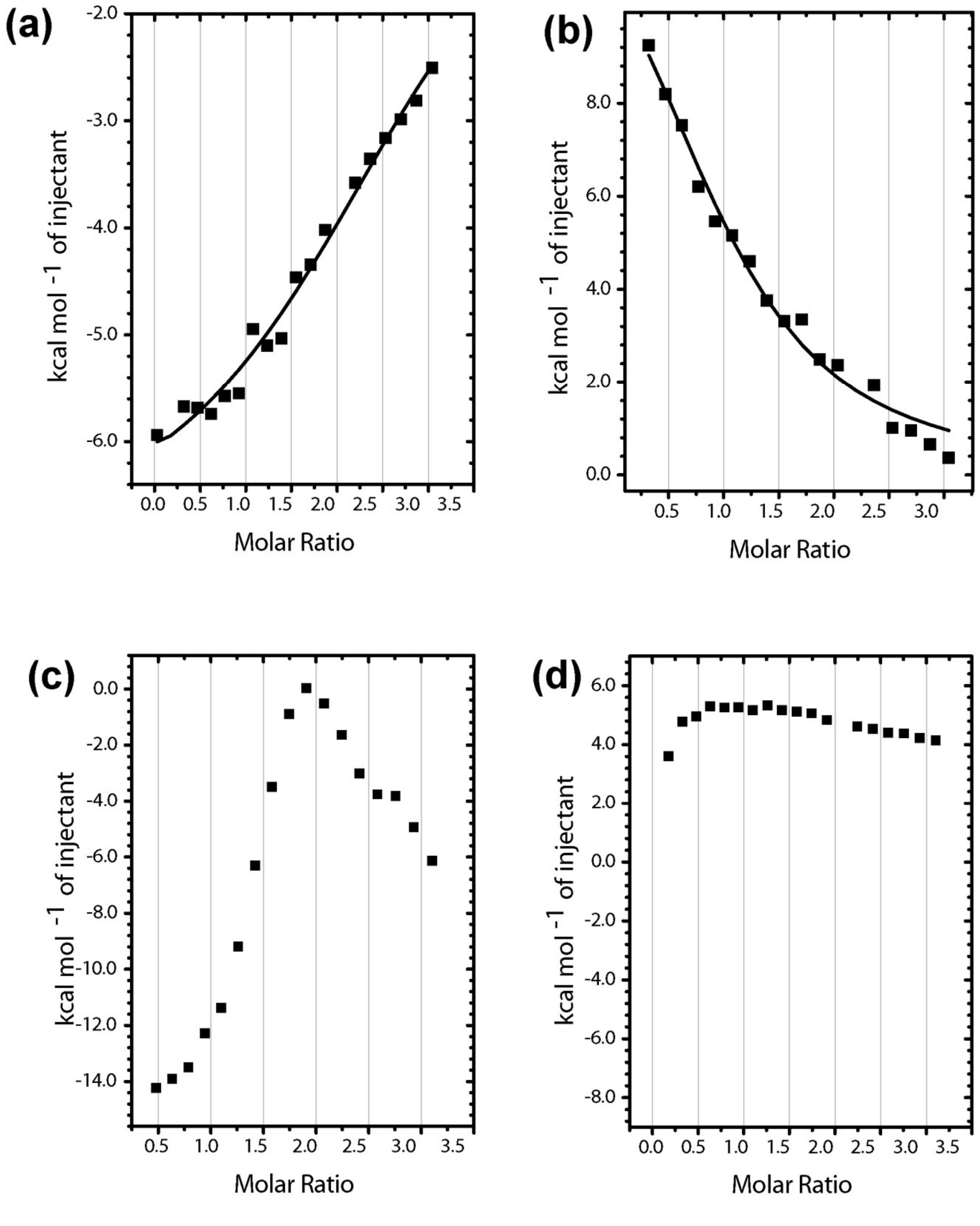

Figure S3. Isothermal titration calorimetry (ITC) data for binding interactions with soluble xyloglucan of (a) CBM3, (b) X2-CBM3,(c) X2, and (d) buffer (control). Binding affinity constants (K_A) and thermodynamic properties are presented in Table 2.

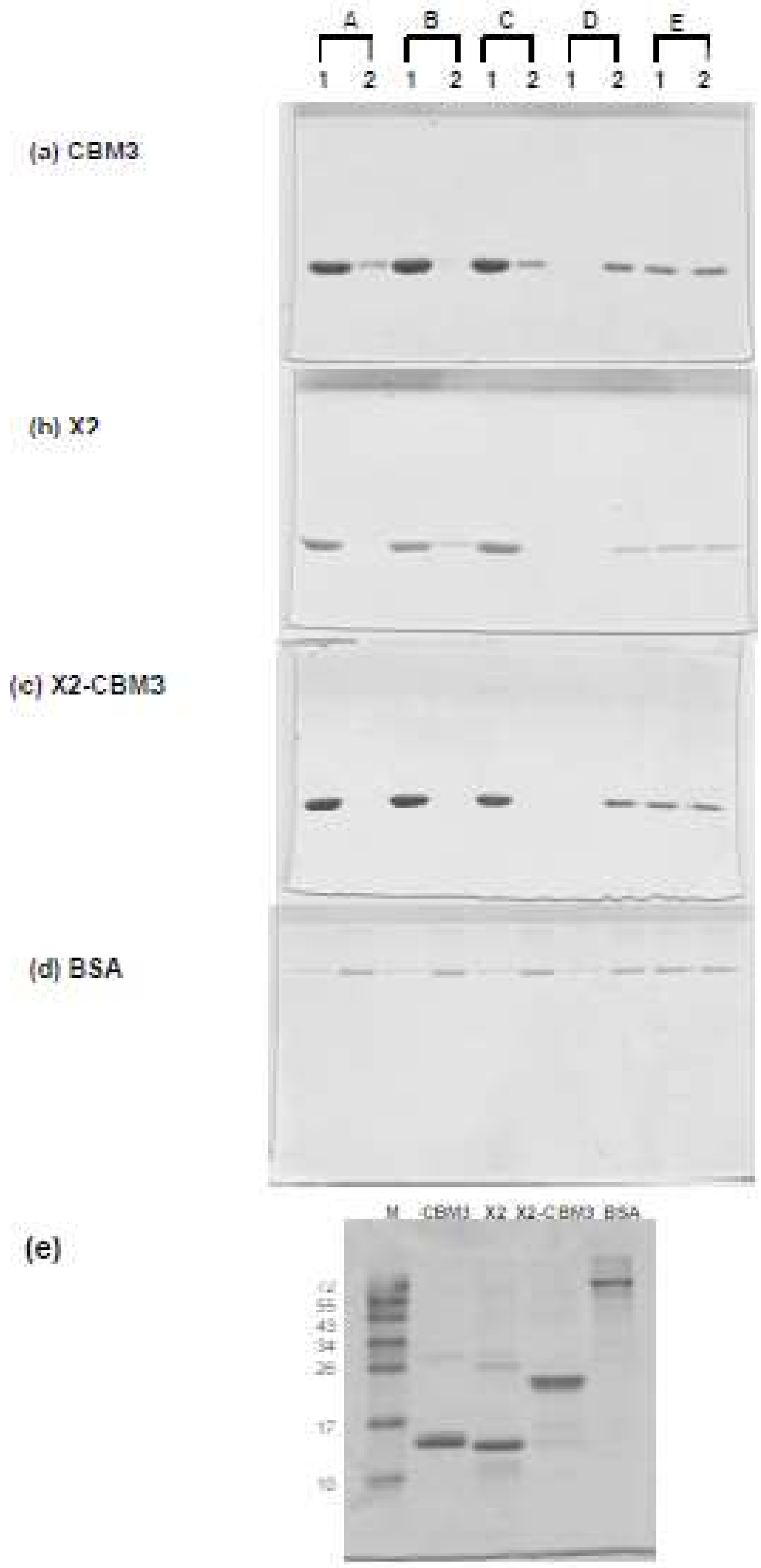

Figure S4. Full-length SDS-PAGE gels for binding assay of X2-CBM3 and its independent modules to insoluble polysaccharides. 10 μg of purified CBM3 (a), X2 (b), X2-CBM3 (c) and BSA (d) was incubated with 4 mg of insoluble substrates, such as (A)

biomass, (B) Avicel, (C) PASC, (D) starch and bound (1) and Unbound*(2) proteins were analysed on SDS-PAGE gel. (E) The same amount of protein used in the binding assay in absence of the polysaccharide was included as a control to observe aggregation over the incubation period. (e) Separation of purified CBM3, X2 and X2-CBM3 on an SDS-PAGE gel along with molecular weight marker (M). *Only 20 µl of 250 µl unbound fraction was loaded on the SDS-PAGE gel.

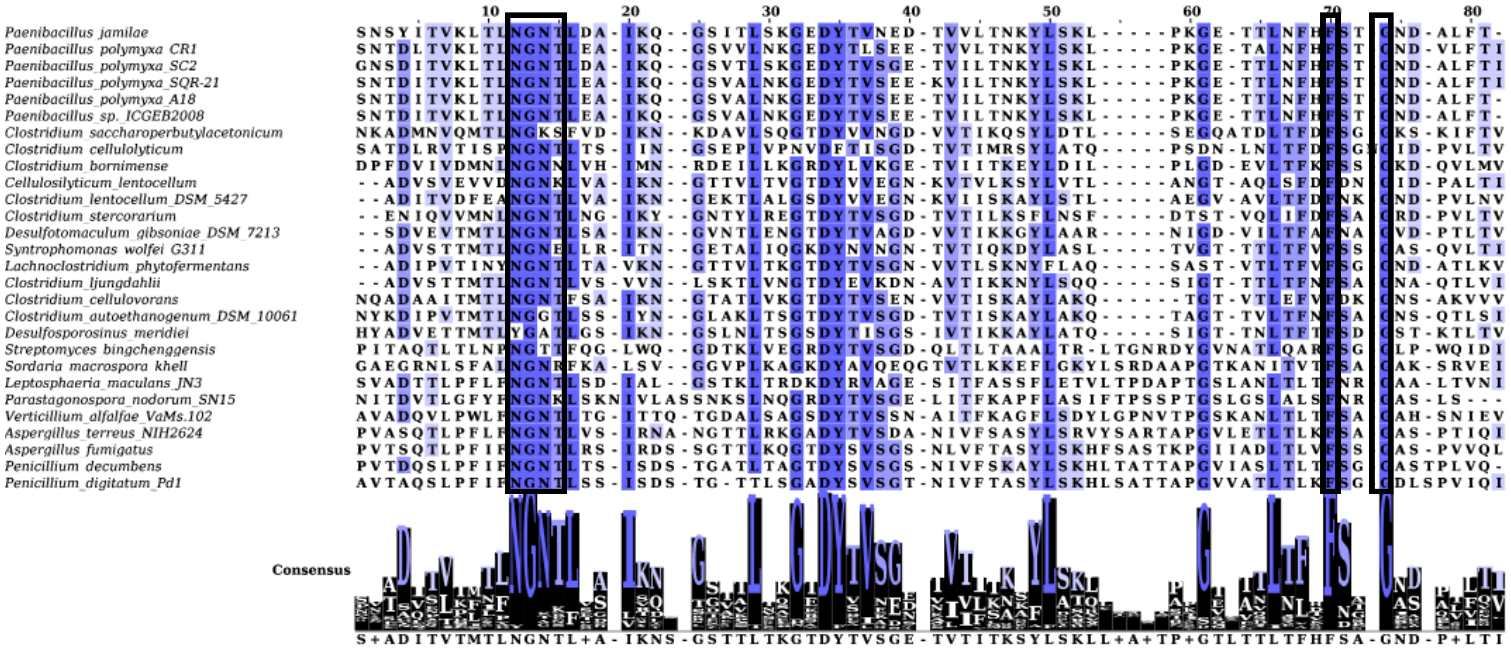

Figure S5. Alignment of module X2 of different organisms. The X2 region was extracted from the PP3 protein of *P. polymyxa* A18 and aligned with X2 of other species using Clustal Omega. Alignment was visualized using Jalview tool. The intensity of blue color defines the degree of conservation. The conserved regions reported earlier[43] have been boxed.

(a)

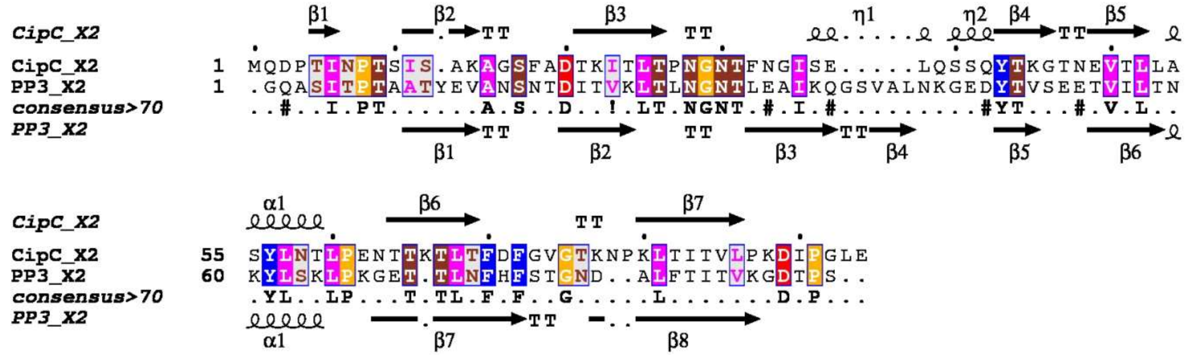

(b)

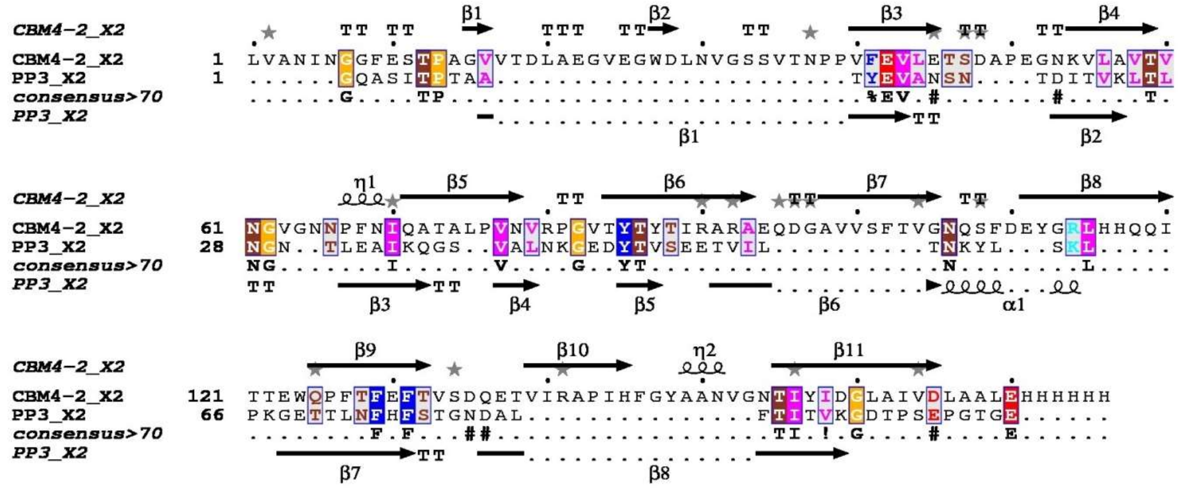

Figure S6. Alignment of X2 of PP3 of *P. polymyxa* A18 with (a) X2 of CipCof *Clostridium cellulolyticum* and (b) CBM4-2 X2-L110F of *Rhodothermus marinus*. The alignment was performed using Clustal Omega. Secondary structural features presented in the alignment are derived from the respective structures of the CipC_X2 module (1EHX), CBM4-2 X2-L110F (4BJ0) and modeled structure of X2 of PP3.

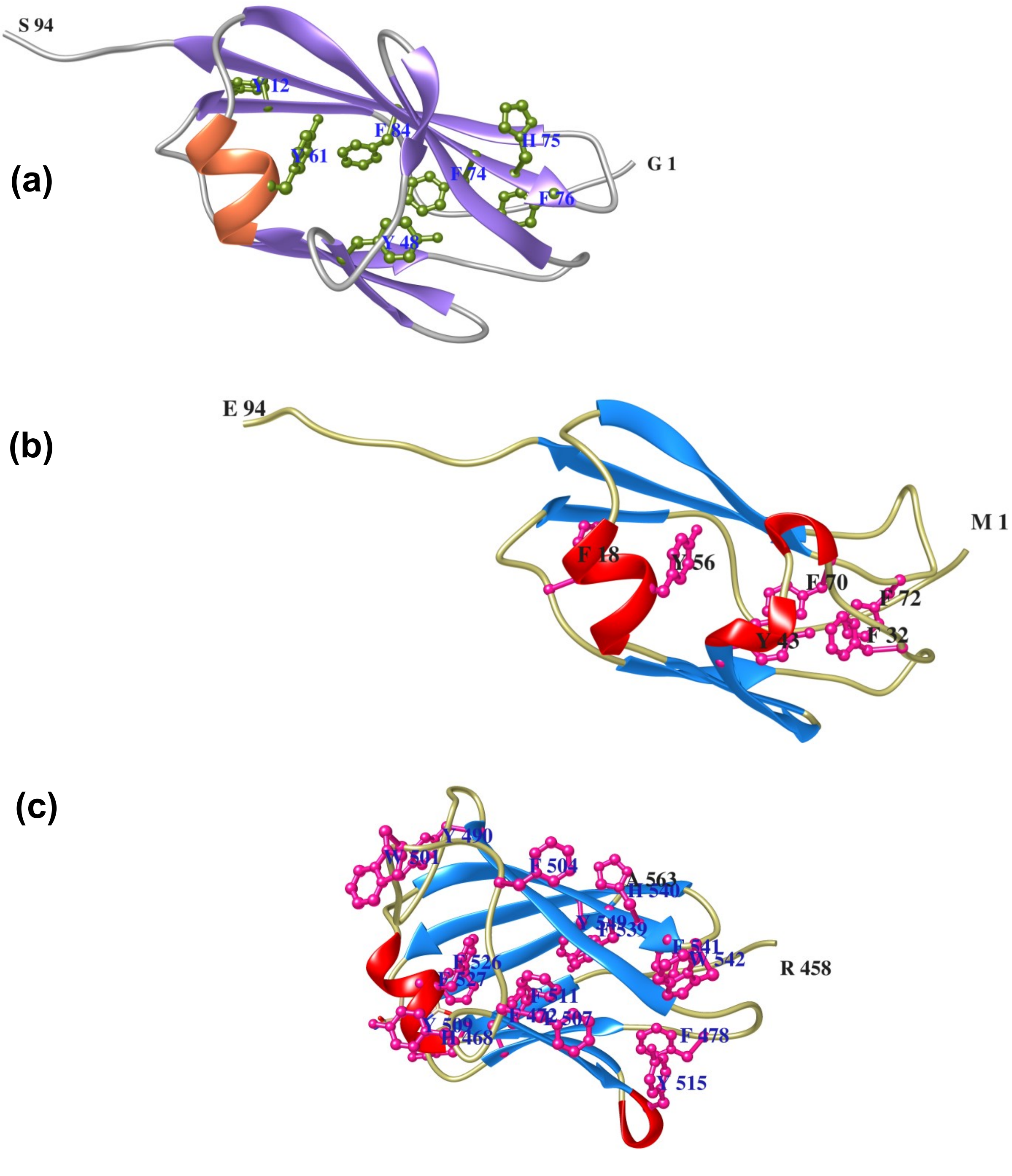

Figure S7. (a) Structural model of *P. polymyxa* A18 PP3-X2 as predicted using MODELLER. Aromatic acid residues and histidine are highlighted in green. **Representations of already known structures (b) of CipC-X2 of *C. cellulolyticum* (PDB ID 1EHX) and (c) of CBM46 of *Bacillus halodurans* (PDB ID 4UZ8).** Aromatic acid residues are highlighted in pink.

Table S1. Annotation of Carbohydrate Binding Modules (CBMs) in *P. polymyxa* A18 genome

NCBI ID	Carbohydrate active enzyme ANnotation<br><br>dbCAN (46)	CAZyme Analysis Toolkit (CAT)		CBM common to all three (19)
		Sequence similarity (121)	Association links with Pfam (103)	
WP_017427084.1		CBM2 CBM10		
WP_016821067.1		CBM12		
WP_038978019.1		CBM5 CBM12		
WP_013372176.1		CBM13	CBM13	
WP_038978237.1		CBM13		
WP_017426909.1	CBM13	CBM13	CBM13	CBM13
WP_017426818.1	CBM16	CBM16	CBM16	CBM16
WP_016818667.1		CBM2		
WP_017425852.1		CBM2		
WP_016821937.1		CBM2		
WP_017425698.1		CBM2		
WP_016818812.1		CBM2		
WP_017427459.1		CBM2		
WP_016821370.1		CBM2 CBM57	CBM2 CBM57	
WP_017426951.1		CBM2		
WP_038978016.1		CBM20		
WP_017427423.1		CBM20	CBM20	
WP_038978282.1		CBM20		
WP_017426140.1	CBM22 CBM22	CBM22		
WP_017426122.1	CBM25 CBM25	CBM25 CBM25	CBM25 CBM25	CBM25 CBM25
WP_017427126.1		CBM3		
WP_017427072.1	CBM3 CBM35	CBM3 CBM35	CBM3 CBM35	CBM3 CBM35
		CBM3	CBM3	
		CBM3		
WP_026065408.1	CBM3	CBM3	CBM3	CBM3
WP_016821398.1	CBM3	CBM3	CBM3	CBM3
WP_017427741.1	CBM3 CBM46	CBM3 CBM_X2	CBM3 CBM_X2	CBM3 CBM_X2[ǂ]
WP_017426252.1		CBM32		
WP_017426987.1		CBM32		
WP_017426618.1		CBM32		
WP_017428382.1		CBM32		
WP_017427689.1	CBM32	CBM32		
WP_017427287.1		CBM32		
WP_017428467.1		CBM32		
WP_017425757.1	CBM32	CBM32		
WP_026065438.1		CBM32		
WP_049885680.1		CBM35		
WP_017428375.1		CBM37		

WP_038978013.1	CBM38 CBM38CBM38	CBM38		
WP_016821615.1		CBM48	CBM48	
WP_017426754.1		CBM48	CBM48	
WP_017426749.1	CBM41 CBM48	CBM41 CBM48	CBM41 CBM48	CBM41 CBM48
WP_038978277.1		CBM41 CBM48		
WP_017427316.1		CBM5		
WP_017427172.1		CBM5		
		CBM5		
WP_017428220.1		CBM50	CBM50	
WP_017426860.1		CBM50	CBM50	
WP_016819801.1		CBM50		
WP_017426357.1		CBM50	CBM50	
WP_017426414.1	CBM50	CBM50		
WP_016821264.1		CBM50	CBM50	
WP_017426474.1		CBM50	CBM50	
WP_016820510.1		CBM50		
WP_017425924.1		CBM50	CBM50	
WP_017425884.1		CBM50	CBM50	
WP_017425862.1		CBM50	CBM50	
WP_017425833.1		CBM50	CBM50	
WP_017427950.1		CBM50	CBM50	
WP_017427429.1	CBM50	CBM50		
WP_019687026.1		CBM50	CBM50	
WP_019687404.1		CBM50		
WP_017425990.1		CBM50	CBM50	
WP_013371461.1		CBM50		
WP_017427132.1		CBM50	CBM50	
WP_016819977.1		CBM50	CBM50	
WP_017425564.1		CBM50	CBM50	
WP_017425953.1		CBM50		
WP_049885702.1		CBM50		
WP_016822129.1		CBM50	CBM50	
WP_017427067.1		CBM50		
WP_017427201.1		CBM50	CBM50	
WP_017427183.1		CBM50	CBM50	
WP_017427890.1		CBM50		
WP_016818546.1		CBM50		
WP_017428105.1		CBM50	CBM50	
WP_017428146.1		CBM50		
WP_016822626.1		CBM50		
WP_026065502.1		CBM54		
WP_017427075.1		CBM54		
WP_017425971.1		CBM54	CBM54	
WP_017425583.1	CBM56	CBM56		
WP_017426776.1	CBM44	CBM57	CBM32	

WP_017426775.1		CBM57		
WP_017428602.1		CBM6		
WP_017428582.1	CBM6	CBM6	CBM6	CBM6
WP_017426918.1	CBM36 CBM36	CBM6 CBM36	CBM36	CBM36*
WP_038978105.1		CBM6 CBM13		
WP_017425732.1		CBM6 CBM13		
WP_017426617.1		CBM6		
WP_017426393.1		CBM6		
WP_038978142.1	CBM66 CBM66CBM66	CBM66		
WP_017426926.1		CBM9 CBM22		
WP_017426884.1	CBM36	CBM36	CBM36	CBM36
WP_026065433.1			CBM50	
WP_016820718.1	CBM48		CBM48	
WP_017427581.1	CBM26	CBM26		
WP_038978275.1	CBM34	CBM34		
WP_017426462.1		CBM32 CBM54		
WP_017426238.1		CBM61		
WP_016822953.1			CBM48	
WP_016822952.1			CBM50	
WP_017427115.1	CBM50	CBM50		
WP_038978027.1		CBM50		
WP_017427585.1			CBM50	
WP_017428011.1	CBM35 CBM35	CBM35	CBM35	CBM35*
	CBM13		CBM13	
WP_017426115.1	CBM32			
		CBM13		
WP_017426982.1	CBM13 CBM32	CBM13 CBM32	CBM13 CBM32	CBM13 CBM32
WP_038978163.1	CBM46	CBM46	CBM46	CBM46
WP_016818773.1	CBM59	CBM59		
WP_017426330.1		CBM13		
WP_017428310.1	CBM67			
WP_017425706.1		CBM35		
WP_017426788.1		CBM13		
WP_017425629.1	CBM66	CBM66		
	CBM3			
WP_017426405.1	CBM50		CBM50	
WP_017428194.1	CBM63		CBM63	
WP_017428191.1			CBM54	
WP_017428236.1			CBM50	
WP_017426799.1			CBM50	
WP_017426188.1			CBM50	
WP_026065382.1			CBM50	
WP_017426510.1			CBM50	
WP_016821162.1			CBM1	
WP_016820787.1			CBM50	

WP_017426609.1			CBM26	
WP_017426615.1			CBM26	
WP_016820500.1			CBM50	
WP_016820774.1			CBM50	
WP_013309551.1			CBM50	
WP_016819622.1			CBM50	
WP_017425853.1			CBM50	
WP_017425807.1			CBM50	
WP_017425798.1			CBM26	
WP_017425766.1			CBM26	
WP_017426022.1			CBM26	
WP_017426045.1			CBM26	
WP_016822700.1			CBM50	
WP_025366109.1			CBM50	
WP_017428643.1			CBM26	
WP_017428614.1			CBM50	
WP_013373475.1			CBM26	
WP_017427137.1			CBM50	
WP_017427258.1			CBM50	
WP_017427272.1			CBM54	
WP_017427303.1			CBM26	
WP_017427331.1			CBM20	
WP_017427332.1			CBM20	
WP_016818619.1			CBM50	
WP_017427402.1			CBM50	
WP_038978223.1			CBM6	
WP_017425569.1			CBM50	
WP_016820244.1			CBM50	
WP_017425689.1			CBM50	
WP_017425661.1			CBM50	
WP_013371107.1			CBM50	
WP_017428031.1			CBM50	
WP_016819467.1			CBM26	
WP_017428066.1			CBM50	
WP_016819369.1			CBM26	
WP_016818882.1			CBM50	
WP_017427239.1			CBM50	
WP_017427223.1			CBM26	
WP_017427216.1			CBM50	
WP_002440835.1			CBM26	
WP_038978284.1			CBM50	
WP_016821500.1			CBM26	
WP_038978006.1			CBM50	

* CBM35 and CBM36 were predicted to be present as tandem repeats by dbCAN, highlighted in light gray.

Table S2. Relative transcript levels of genes coding for CBM containing polypeptides in the presence of different carbon substrates

CBM containing Polypeptide			Biomass		Avicel		CMC		Xylan	
PP	CBM	GH	Fold change*	P value**	Fold change*	P value**	Fold change*	P value**	Fold change*	P value**
1	3	GH5	1.00	0.0299	-1.32	0.0320	1.53	0.0189	6.89	0.0022
2	3	GH6	7.68	0.0001	9.03	0.0001	6.36	0.0001	1.49	0.0403
3	3, X2	GH74	39.92	0.0001	28.68	0.0003	2.56	0.0127	20.37	0.0001
4	3, 35	GH26, GH44	1.95	0.0079	1.76	0.0171	4.77	0.0033	1.73	0.0408
5	16	PL9	-2.13	0.0235	-7.25	0.0001	-1.41	0.0296	2.51	0.1904
6	46	GH5	4.65	0.0375	6.42	0.0308	1.73	0.0206	11.57	0.0055
7	6	GH3	1.75	0.3595	-1.62	0.0098	1.76	0.0175	6.59	0.0001
8	36, 36	GH43	8.17	0.0006	2.23	0.0485	-1.23	0.4203	-1.15	0.2543
9	13, 32	GH5	-1.01	0.8865	-1.38	0.0018	1.74	0.0001	1.64	0.0293
10	13	PL5	1.44	0.0171	1.10	0.0167	5.04	0.0014	5.23	0.0005
11	36	CE4	-3.15	0.0146	1.24	0.0392	67.51	0.0001	3.00	0.0240
12	35, 35	GH26	1.62	0.0378	-2.63	0.0425	-1.35	0.1783	-1.22	0.3410
13	25, 25	GH14, GH13	1.27	0.8166	-1.26	0.1454	36.12	0.0120	60.60	0.0058
14	48, 41	GH13	1.35	0.0071	-1.67	0.0025	8.44	0.0352	3.57	0.0076

*Fold change was calculated with respect to expression of the same gene in cells grown without any carbon substrate and normalized on 16S rRNA.

**Statistical analysis was performed using an independent Student's *t*-test. P-values <0.05 were considered significant.

Table S3. Comparison of xyloglucanase activity of full-length and truncated derivatives towards soluble and insoluble substrates

Substrate	Encoded domain(s)	Specific activity (IU/mg)	Molar specific activity (IU/µmol)	Fold change
Xyloglucan (soluble)	GH74	2.4 (±0.18)	193.47 (±11)	1
	GH74-X2	3.47 (±0.11)	304.49 (±31.23)	1.57
	GH74-CBM3	3.45 (±0.26)	308.43 (±18.76)	1.6
	GH74-X2-CBM3	4.58 (±0.17)	489.79 (±56)	2.5
Ammonium hydroxide-treated biomass (insoluble)	GH74	0.01 (±0.012)	3.20 (±1.71)	1
	GH74-X2	0.02 (±0.003)	7.06 (±1.29)	2.2
	GH74-CBM3	0.019 (±0.009)	6.91 (±1.12)	2.1
	GH74-X2-CBM3	0.034 (±0.001)	14.71 (±0.62)	4.6

Table S4. List of primers used for RT-qPCR

Polypeptide	Forward oligo sequence	Reverse oligo sequence	Annealing temperature
1	GCCAAGGATAATGCGAT	GAAATTTCTACATATTTATC	43
2	GTAATGTATCGTGCTGG	CCAATTTCAATATAGTTG	41
3	GCAACAAGTAATACGATC	CAATTTCTGCATAAGAATC	45
4	GGTGAACGATAATCACCT	CTGACTTCCAAATAATAATC	47
5	CCCGGAGAAGATGGTAT	AGCATACACTGTTGCCC	47
6	AGGATACCAAGGTCACT	CGACGATTTTAAAATTCC	43
7	GGCGTAGACATGAATACG	TTCTCCACTAAGTGACAG	47
8	CATAATATCTCAGTGCG	GAAAATTCAATAAAATCC	39
9	TAGCCGGAGGCTCCTCA	CTTCCACTTCTGCTGATC	49
10	ACAAATGGCTCTCTTGATC	CCAAGCCTCCATTGTTGA	49
11	CATCATTTTTCACTCCGT	GTTGATCTCCAAATAGTC	45
12	AATGCACCTTCAAGCGGG	CTTGACGTAGTCGATATG	47
13	GTATACTACAAAAAAGGC	CCAGTGCTGAAAAGGTA	45
14	TATGGGGCACCGGACAC	ATATTCCTCTCTGCCACT	47
Reference gene-16S	CCCACCTTCCTCCGGTTT	GCAACGCGAAGAACCTTAC	53
Non coding region1 (NC1)	ATAAGTGACCTGTTCAT	CCGATTTATGAATCTTTA	41
Non coding region2 (NC2)	GCTGCGAAAGCGTAGTT	TAACTTGTTCGGAGTGTT	45

Table S5. Strains, plasmids, and primers used in the study

Name	Description	Reference
**Strains**		
Paenibacillus polymyxa A18	Native cellulolytic bacterium isolated from termite gut	22
Escherichia coli DH5α	F^- Φ80*lac*ZΔM15 Δ(*lac*ZYA-*arg*F) U169 *rec*A1 *end*A1 *hsd*R17(r_k^-, m_k^+) *pho*A*sup*E44 *thi*-1 *gyr*A96 *rel*A1 λ^-	Life Technologies
**Plasmids**		
pQE-PP3-1	Xyloglucanasewithout signal peptide expressed under T5 promoter	This study
pQE-PP3-4	GH74 under expressed T5 promoter	This study
pQE-PP3-2	GH74 catalytic domain with X2 domain expressed under T5 promoter	This study
pQE-PP3-3	GH74 catalytic domain with CBM3 expressed under T5 promoter	This study
pQE-X2	X2 domain expressed under T7 promoter	This study
pQE-CBM3	CBM3 domain expressed under T7 promoter	This study
pQE-X2-CBM3	X2-CBM3 domain expressed under T7 promoter	This study
pQE–Endo5A-GS-CBM	Endoglucanase gene fused with X2-CBM3 with linker in between expressed under T7 promoter	This study
pQE–Xyl11D-GS-CBM	Xylanase gene fused with X2-CBM3 with linker in between under T7 promoter	This study
**Primers**		
Xylg_F	CGTGCATGCCCAAGTGACGATTATACTTG	This study
Xylg_R	CGCGTCGACTTAAGGCTCAATTCCCCATT	This study
GH74_R	CGTGTCGACCTATGCTTGCCCGGTAGATG	This study
GH74_R_kpnI	CGTGGTACCCGACCCACCACCGCCCGAGCCACCG TGCTTGCCCGGTAGATGG	This study
X2_F_sacI	CGCGAGCTCAGTATTACGCCGACGGCTG	This study
X2_F_kpnI	CGCGGTACCAGTATTACGCCGACGGCTG	
X2_R_salI	CAAGTCGACCTA CTGTTCCCGGCTCACTTGG	This study
CBM3_F_sacI	AATGAGCTCGCGAGGGTGCTCTGAAG	
CBM3_F_kpnI	AATGGTACCGCGAGGGTGCTCTGAAG	This study
CBM3_R_salI	CGTGTCGACCTAACCAATTTCTGCATAAGAA	This study

Note: The restriction enzyme sites are underlined.
